# New 2-Oxoindolin Phosphonates as Novel Agents to Treat Cancer: A Green Synthesis and Molecular Modeling

**DOI:** 10.3390/molecules23081981

**Published:** 2018-08-08

**Authors:** Shailee V. Tiwari, Nawaz S. Sharif, Rekha I. Gajare, Julio A. Seijas Vazquez, Jaiprakash N. Sangshetti, Manoj D. Damale, Anna Pratima G. Nikalje

**Affiliations:** 1 Y. B. Chavan College of Pharmacy, Dr. Rafiq Zakaria Campus, Rauza Baug, Aurangabad, Maharashtra 431001, India; shailee2010@gmail.com (S.V.T.); nawajsharifsk@gmail.com (N.S.S.); rgajare3@gmail.com (R.I.G.); jnsangshetti@rediffmail.com (J.N.S.); 2 Departamento de Química Orgánica, Facultad de Ciencias, Universidad of Santiago De Compostela, Alfonso X el Sabio, 27002 Lugo, Spain; julioa.seijas@usc.es; 3 Department of Pharmaceutical Medicinal Chemistry, Srinath College of Pharmacy, Bajajnagar Aurangabad, Maharashtra 431136, India; pharmalink1985@gmail.com

**Keywords:** indole-2,3-dione, α-aminophosphonates, in-vitro anticancer activity, ceric ammonium nitrate, docking

## Abstract

The work reports the facile synthesis of novel α-aminophosphonate derivatives coupled with indole-2,3-dione moieties, namely the diethyl(substituted phenyl/heteroaryl)(2-(2-oxoindolin-3-ylidene)hydrazinyl)methylphosphonates derivatives **4**(**a**–**n**). One-pot three component Kabachnik-Fields reactions were used to synthesize these derivatives. The reaction was carried out at room temperature by stirring in presence of ceric ammonium nitrate (CAN) as a green catalyst. The structures of the synthesized compounds were established by spectral studies. The synthesized derivatives **4**(**a**–**n**) were evaluated for their in vitro anticancer activity against six human cancer cell lines by the SRB assay method. The cancer cell lines used in this research work are SK-MEL-2 (melanoma), MCF-7 (breast cancer), IMR-32 (neuroblastoma) MG-63 (human osteosarcoma), HT-29 (human colon cancer) and Hep-G2 (human hepatoma). All the synthesized derivatives inhibited the cell proliferation. Importantly, all the target compounds showed no cytotoxicity towards normal tissue cells (GI_50_ > 250 µM). A docking study was performed to predict the mode of action. Docking results indicate that the compounds have good binding with the enzyme tyrosine kinase as well as with microtubules, which makes them dual inhibitors. The result of in-silico bioavailability studies suggests that the compounds from the present series have good oral drug-like properties and are non-toxic in nature. In vivo acute oral toxicity study results indicate that the compounds can be considered safe, and therefore could be developed in the future as good anticancer agents or as leads for the design and synthesis of novel anticancer agents.

## 1. Introduction

The number of patients dying across the globe because of cancer and the non-availability of effective, non-toxic anticancer drugs in the present drug market continues to increase. Consequently, preventing this fatal disease is more challenging and hence the invention of novel anticancer agents is of paramount significance.

During carcinogenesis, an angiogenic switch occurs and several angiogenic growth factors stimulate their receptor tyrosine kinases (RTKs) to initiate multiple pro-angiogenic events [1]. A therapeutic strategy to inhibit these key angiogenic proteins or their RTKs was envisioned [2,3,4]. Multiple inhibitors targeting the different types of RTKs receptors have been studied by scientists all over the world to synthesize target-oriented drug molecules. Epidermal growth factor receptor (EGFR), vascular endothelial growth factor (VEGF) and/or platelet-derived growth factor receptor (PDGFR-2) are now used clinically. These RTKs are noted to have multi-kinase effects [5], and this appears to be imperative for improved anticancer activity.

The most successful anticancer drugs in clinical use acting by inhibiting tubulin polymerization are vincristine, vinblastine, vindesine, etc. [6]. The destabilizing agents bind to tubulin at different binding sites, including the vinca domain and the colchicines site [6].

Combination cancer chemotherapy is not a new idea. Recent studies indicate that the combination of antiangiogenic agents with cytotoxic agents is more effective in cancer treatment [7]. Examples of RTK inhibitors as the anti-angiogenic component along with cytotoxic chemotherapeutic agents [8,9] are lapatinib with carboplatin, paclitaxel and trastuzumab used in metastatic breast cancer [10,11] and docetaxel, gemcitabine and pazopanib as a treatment for soft tissue sarcoma [12], etc. The advantage of combination chemotherapy, particularly with RTK inhibitors, is reduced redundancy [9]. It is also beneficial when RTK inhibitors are combined with conventional cancer therapeutics [8,9]. The RTK inhibitors are cytostatic in nature and the tubulin inhibitors are cytotoxic in nature. If the scientists were to design the drug that contains a pharmacophore which hascytotoxic plus cytostatic pharmacological properties, then such a drug could be most promising in treating cancer patients. In keeping with the principles of combination chemotherapy [8,9], such single entities would act at a time at two or more distinct targets. Combination chemotherapy can prevent or delay the emergence of resistance, avoid drug–drug interactions, and circumvent pharmacokinetic problems and overlapping toxicities. Therefore, we thought to combine RTK inhibitory and cytotoxic activities in a single molecule to afford combination chemotherapeutics via a single agent [13,14].

The indole scaffold can be a keystone to discover drug-like kinase inhibitor molecules. Variation of substituents on the indole scaffold may have a great impact on the pharmacokinetic and pharmacodynamic behavior of the resultant molecule [15]. Tyrosine kinases are enzymes responsible for the activation of many proteins by signal transduction cascades. A pharmaceutical drug that inhibits tyrosine kinases is tyrosine kinase inhibitor (TKI) [16].

α-Aminophosphonates are among the most studied bioactive organophosphorus derivatives. They are used as enzyme inhibitors [17], inhibitors of serine hydrolase [18], peptide mimics [19], antiviral [20], antibacterial [21], antifungal [22], anticancer [23], anti-HIV [24], antibiotics [25], herbicidal [26] agents, etc. Indoles possess various medicinal properties like antibacterial, antifungal, anti-malarial, anticonvulsant and anti-inflammatory effects, etc. [27].

Isatin, chemically known as 1-*H*-indole-2,3-dione, and its derivatives possess a broad range of pharmacological properties. They are extensively utilized as a starting material for the synthesis of a broad range of heterocyclic compounds and as substrates for drug synthesis. In terms of its mode of action, isatin itself is proposed to inhibit cancer cell proliferation via interaction with extracellular signal-related protein kinases (ERKs), thereby promoting apoptosis. These compounds inhibit cancer cell proliferation and tumor growth via interaction with a variety of intracellular targets such as DNA, telomerase, tubulin, P glycoprotein, protein kinases and phosphatases [28]. Isatin-based hydrazones have been identified as inhibitors of the protein tyrosine phosphatase Shp2 [29]. The protein tyrosine phosphatase Shp2 plays a key role in cell signaling, cell proliferation, differentiation and migration [30].

**Figure 1:**
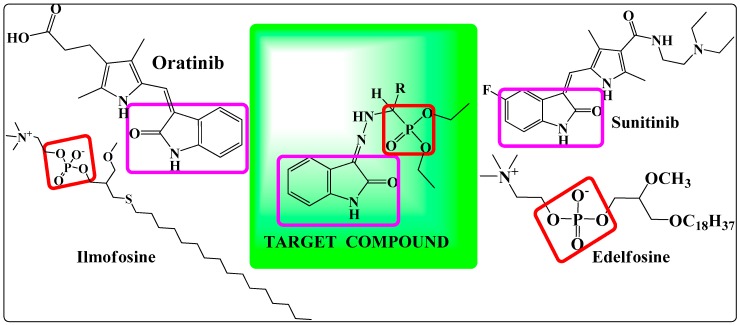
Designing protocol for target compounds **4**(**a**–**n**).

The design protocol for the target molecules is revealed in Figure 1. Marketed anticancer drugs such as sunitinib [31] and oratinib contain a 2-oxoindolin-3-ylidene moiety. Ilmofosin and Edelfosin contain a phosphonatemoiety. A recently marketed anticancer drug, toceranib phosphate [32] contains a 2-oxoindol-3-ylidene as well as a phosphonate moiety. The biological importance of the 2-oxoindolin-3-ylidene scaffold and α-aminophosphonates and the ongoing interest of our research group [16,33,34,35,36] in the synthesis of anticancer agents encouraged us to synthesize coupled derivatives containing isatin-based hydrazones and α-aminophosphonates with the hope of obtaining novel hybrid derivatives with better anticancer activity and minimal toxicity.

The one pot three-component reaction of aromatic/heterocyclic aldehydes, amines and triethylphosphite, also known as the Kabachnik–Fields reaction [37] was performed using various catalysts like Cu(OTf)_2_[38], VB1 [39], Al(OTf)_3_ [40], ZrOCl_2_·8H_2_O [41], YbCl_3_ [42], lanthanide triflates [43], Mg(ClO_4_)_2_ [44], LiClO_4_[45] etc. The Kabachnik–Fields reaction was also promoted in the presence of ceric (IV) ammonium nitrate (CAN) as a green catalyst. CAN catalyst has advantages like the very small amount of this catalyst is needed to complete reactions in most of the cases, lower costs, ecofriendly nature, high reactivity, non-toxicity, and ease of handling [46].

Fourteen new diethyl (substituted phenyl/heteroaryl)(2-(2-oxoindolin-3ylidene)hydrazinyl)methyl phosphonates derivatives **4**(**a**–**n**) were synthesized at room temperature. The synthesized compounds were screened for their in-vitro anticancer activity against six human cancer cell lines by the SRB assay method. The cancer cell lines used in this research work are SK-MEL-2 (melanoma), MCF-7 (breast cancer), IMR-32 (neuroblastoma), MG-63 (human osteosarcoma), HT-29 (human colon cancer) and Hep-G2 (human hepatoma). All the synthesized derivatives **4**(**a**–**n**) were also tested for their cytotoxic effects on normal cell lines i.e., NIH/3T3 (murine embryonic fibroblast) by the SRB assay method. The results showed that the synthesized compounds inhibited the proliferation of these selected cancer cell lines at moderate to high rates. In order to explore the anticancer activity of the designed derivatives they were subjected to a computational molecular docking study. The synthesized compounds that demonstrated potential in vitro anticancer activities were further screened for their in vivo acute oral toxicity study and gross behavioral studies using Swiss albino mice.

## 2. Results

### 2.1. Chemistry

**Scheme 1:**
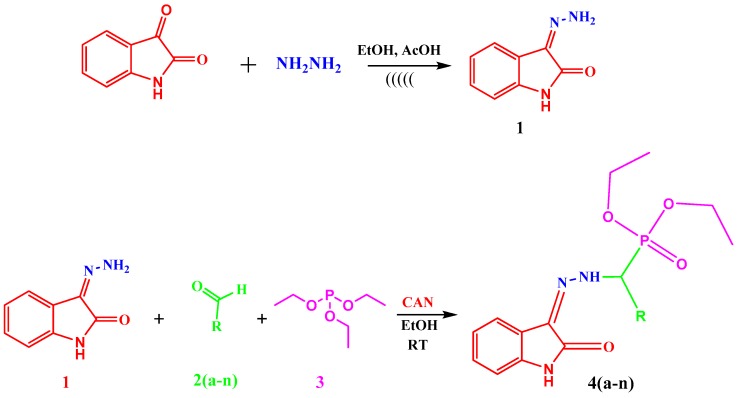
One pot three component synthesis of diethyl (substituted phenyl/heteroaryl)(2-(2-oxoindolin-3ylidene)hydrazinyl)methyl phosphonates derivatives **4**(**a**–**n**).

The diethyl (substituted phenyl/heteroaryl)(2-(2-oxoindolin-3ylidene)hydrazinyl)methyl phosphonate derivatives **4**(**a**–**n**) were synthesized as summarized in Scheme 1. 3-Hydrazonoindolin-2-one (**1**) was synthesized by reacting indole-2,3-dione (isatin) with hydrazine hydrate in methanol in the presence of glacial acetic acid as a catalyst by the conventional method using molecular sieves. 3-Hydrazonoindolin-2-one (**1**) was also synthesized by an ultrasonication-assisted green method by replacement of methanol with ethanol.

α-Aminophosphonate derivatives **4**(**a**–**n**) were synthesized by the Kabachnik-Fields method by reacting 3-hydrazonoindolin-2-one (**1**), substituted aldehydes **2**(**a**–**n**) and triethylphosphite (**3**) via a one pot synthetic step in the presence of CAN as a green catalyst. CAN activates the imine formation due to which addition of phosphite to furnish a phosphonium intermediate is facilitated. This phosphonium intermediate reacts with water to give the title compounds **4**(**a**–**n**). The mechanism of synthesis is as shown in Figure S1 in the Supplementary File.

The diethyl (substituted phenyl/heteroaryl)(2-(2-oxoindolin-3ylidene)hydrazinyl)methyl phosphonates derivatives **4**(**a**–**n**) were thus synthesized using the multicomponent reactions (MCRs). The α-Aminophosphonate derivatives **4**(**a**–**n**) can be synthesized using the multicomponent reaction (MCR) concept or by using multistep reactions. In the multistep reactions first 3-hydrazonoindolin-2-one (**1**) and substituted aldehydes **2**(**a**–**n**) are allowed to react to give a Schiff base intermediate which is extracted and used for next step. In the next step the Schiff base intermediate is allowed to react with triethylphosphite (**3**) to give the final title compounds **4**(**a**–**n**). In our research work we have carried out one pot synthesis in presence of CAN as green catalyst which makes our synthetic route green.

All the synthesized compounds were characterized and confirmed by spectral analysis like; FTIR, ^1^H-NMR, ^13^C-NMR, ^31^P-NMR, MS and elemental analysis. The purity of the synthesized compounds was determined by thin layer chromatography (TLC). The melting points were determined in open capillary tubes and are uncorrected. Physical constants data and the time required for completion of reactions for the diethyl (substituted phenyl/hetery)(2-(2-oxoindolin-3-ylidene)hydrazinyl)methylphosphonate **4**(**a**–**n**) are summarized in Table 1.

### 2.2. In Vitro Anticancer Screening

The in vitro anticancer activity of novel series of α-aminophosphonate derivatives **4**(**a**–**n**), was evaluated by SRB assay against six human cancer cell lines. The human cancer cell lines used are MCF-7, IMR-32, SK-MEL-2, MG-63, HT-29 and Hep-G2. Adriamycin was used as the positive control. The results obtained are summarized in Table 2. All the synthesized derivatives **4**(**a**–**n**) were also tested for their cytotoxic effects on normal cell lines i.e., NIH/3T3 (murine embryonic fibroblast) by the SRB assay method.

From the in vitro anticancer screening data the substitution of a 4-hydroxy-3-methoxyphenyl moiety in compound **4g** resulted in good inhibitory activity against SK-MEL-2 with a GI_50_ value of 24.0 µM. The compounds **4b**, **4f**, **4h** and **4e** show moderate in vitro anticancer activity against SK-MEL-2 with GI_50_ values of 41.4 µM, 51.6 µM, 51.0 µM and 55.2 µM, respectively, while the others show less activity. The 4-chlorophenyl moiety in compound **4b** exhibited good inhibitory activity against MCF-7, with a GI_50_ value of 67.2 µM. The 4-hydroxy-3-ethoxyphenyl moiety in compound **4h** exhibited good inhibitory activity against MCF-7 with a GI_50_ value of 70.7 µM. All the novel synthesized compounds were found to have moderate in vitro anticancer activity for IMR-32 when compared to the standard drug Adriamycin.

All the synthesized derivatives **4**(**a**–**n**) were found to be potent anticancer agents against the MG-63 (human osteosarcoma) cell line, with GI_50_ values similar to that of Adriamycin i.e., the standard drug. The compounds **4d**, **4e**, **4f**, **4i**, **4j**, **4l**, **4m** and **4n** have shown equipotent anticancer activity to that of adriamycin employed as the standard drug against the HT-29 (human colon cancer) cell line. The compounds **4i** and **4l** showed equipotent anticancer activity to that of standard drug Adriamycin against the Hep-G2 (human hepatoma) cell line.

The induction of apoptosis by chemotherapeutic agents has always been a favorite choice in developing anti-cancer therapeutics. To find out whether the treatment with the novel synthesized compounds could lead to loss of cell viability and induction of apoptosis, the MCF-7, IMR-32 SK-MEL-2, MG-63, HT-29 and Hep-G2 cancer cell lines were treated with the GI_50_ concentrations of the novel synthesized compounds **4**(**a**–**n**). Cell morphology was observed at the GI_50_ concentration of the synthesized compounds **4**(**a**–**n**) and photographs were taken under a Nikon Eclipse Ti-S Inverted Research Microscope and the images were processed using the NIS-Elements software. The images of the in vitro anticancer activity of the active compounds from the synthesized compounds **4**(**a**–**n**) on the MCF-7, IMR-32, SK-MEL-2, MG-63, HT-29 and Hep-G2 cancer cell lines are shown in Figures S2–S7, respectively, in the Supplementary File. At the GI_50_ concentration of the potent novel compound **4f** there distinguishing morphological changes were observed in IMR-32 cancer cells such as cell detachment, cell wall deformation, cell shrinkage and reduced number of viable cells in contrast to control cells, which can be clearly observed in Figure S3. It can be concluded from Figure S4 that at the GI_50_ concentration of the most active compound **4g** there were distinctive morphological changes such as cell detachment, cell wall deformation, cell shrinkage and reduced number of viable cells in SK-MEL-2 cancer cell lines in comparison to control cells.

The synthesized compounds **4**(**a**–**n**) showed no cytotoxicity towards normal tissue cells. It’s very vital for cancer treatment that the anticancer drugs have properties of high efficiency and low toxicity. The synthesized compounds **4**(**a**–**n**) were found to be selective towards cancer cells since they did not exhibit cytotoxicity even at GI_50_ > 250 μM on normal tissue cells.

### 2.3. Docking Study

A molecular docking study was carried out in order to find the anticancer activity potential of the synthesized compounds. The synthesized compounds have an indole nucleus in their structure which is similar to that present in anticancer drugs such as vincristine, vinblastine and sunitinib. Sunitinib acts by inhibition of tyrosine kinase therefore, a docking study was carried out using tyrosine kinase. Vincristine and vinblastine bind to the microtubular proteins of the mitotic spindle, leading to crystallization of the microtubule and mitotic arrest or cell death. Therefore, a docking study was carried out using microtubules. In the present study the synthesized compounds are also evaluated for their in-vitro anticancer activity against SK-20 MEL-2 (melanoma), MCF-7 (breast cancer), IMR-32 (neuroblastoma) MG-63 (human osteosarcoma), HT-29 (human colon cancer) and Hep-G2 (human hepatoma) cell lines and based on this assumption synthesized compounds were docked against microtubules and tyrosine kinases (TRKs) to determine their possible mode of inhibition.

In order to identify and analyse binding affinity, binding mode and molecular interactions of the synthesized compounds in active site of receptor molecular docking study was carried out against microtubules and tyrosine kinases (TRKs). Tyrosine kinases (TRKs) are indispensable for numerous processes in the cell. These enzymes catalyze phosphorylation of different cellular substrates. Phosphorylation in turn regulates various cellular functions. Normally, their activity is stringently regulated. However, under pathological conditions, TRKs can be deregulated, leading to alterations in the phosphorylation and resulting in uncontrolled cell division, inhibition of apoptosis, and other abnormalities and consequently to diseases. Inhibition of TRKs has been shown to be a promising therapeutic strategy. Tubulin α and β heterodimer polypeptide chains are found to be an important drug target in breast cancer treatment [47]. Tubulin heterodimer polypeptide chains of α and β tubulin (50 kDa each in size) are the basic structural components of microtubules which are hollow tubes of approximately measure about 25 nm in diameter. Microtubules is important component of cell which is cytoskeletal polymers involved in various cellular functions like as mitosis, organization of intracellular structure and intracellular transport, as well as ciliary and flagellar motility. The α and β heterodimers are very significant for cellular process. In *Homosapiens*, there are six α-tubulin and seven β-tubulin isotypes identified and in the molecular expression of each isotype studied it is found that the molecular expression varies in different tissues and cells [48,49,50]. It is found that tubulin-binding drugs have different affinities for different isotypes of α and β, which affects the overall efficacy in different cancers. There are chemically diverse classes of compounds that bind to the tubulin–microtubule hetrodimer system. Tubulin-binding agents are potent mitotic poisons [51,52]. The literature reports of types of compounds that are synthesized in this project and the result of anticancer activity obtained prompted us to take up of docking study against two targets i.e., TRKs and tubulin inhibitors.

Depending upon the binding affinity, docking score −Log (ki), molecular interaction values given in Table 3. The methyl phosphonate derivatives such as **4f**, **4g**, **4d** and **4e** that show docking scores in between 6.031 to 5.333 are found among the most active ones. The compounds **4a**, **4b**, **4h**, **4c** and **4j** are moderately active, with docking scores of 5.111 to 4.511. The compound **4i** is the least active amongst the entire series of synthesized compounds, with a docking score 3.599.

**Figure 2:**
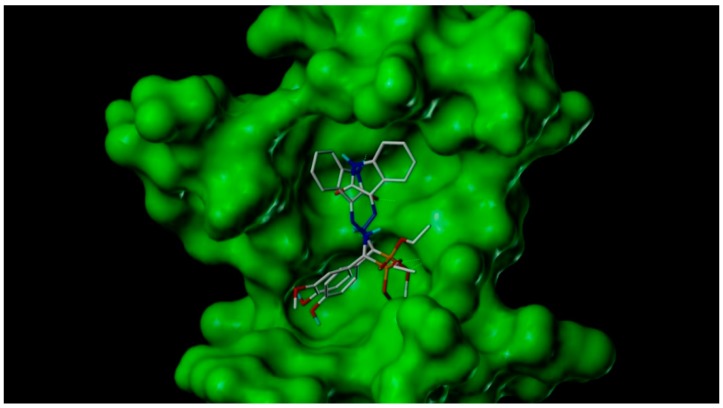
3D representation of binding modes of most active forms of methylphosphonate derivatives **4f** and **4g** in the putative active site of TRKs enzyme.

**Figure 3:**
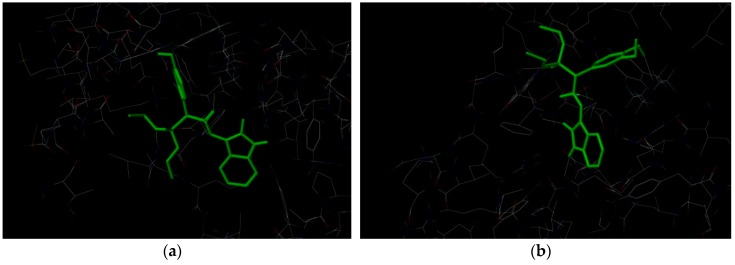
3D representation of binding modes and molecular interactions of methylphosphonate derivatives (**a**): **4f** and (**b**): **4g**.

The most active compounds **4f** against IMR-32 showed overall a very efficient binding mode and penetration of the active site cavity by forming various interactions with active site residues such as ILE758, VAL654, THR870, LYS870, CYS678, and ASP818. The active site residue ILE758 formed hydrogen bond interactions with hydrazine groups with a distance of 1.65 Å. The amino acid CYS678 forms the hydrogen bond interactions with the hydroxyl group of the aryl ring with a distance of 3.15 Å. The amino acids LYS870 and VAL654 form the H bond interactions with the oxoindolinene ring and phosphonate group with distances of 2.25 and 2.75 Å as shown in Figure 2 and Figure 3.

The molecular docking study was also carried out into the active site of tubulin (PDB ID: 1SA0). The molecular docking data against tubulin are shown in Table 4.

Among all the synthesized phosphonate derivatives **4l**, **4f**, **4m** and **4n** have highest potential of inhibitory activity compared the other synthesized derivatives and the standard ADR against the cancer cell lines MG-63, HT-29 and HEP-G2. The other synthesized derivatives also have shown very good inhibitory and binding interactions, indicated in the form of the GI_50_ value, low total docking score, polar score and high crash score data shown in Table 4.

**Figure 4:**
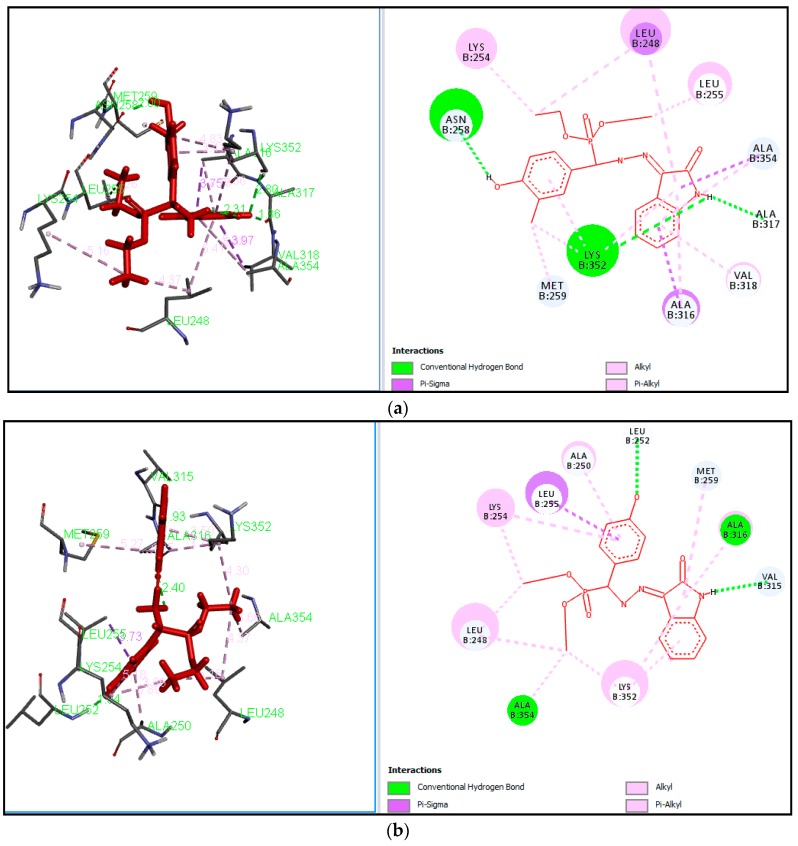
(**a**) Binding pose and molecular interactions of **4l** into the active site of tubulin (**PDB ID: 1SA0**); (**b**) Binding pose and molecular interactions of **4f** into the active site of tubulin (**PDB ID: 1SA0**).

The most active phosphonate derivatives **4l** (6.1242) and **4f** (5.2831) have shown efficient binding mode and penetrating active site cavity in tubulin (1AS0) by forming hydrogen bond interactions with active site residues such as LEU252, ASN258, ALA316, ALA317, LYS352, ALA354, MET259, LEU248, ALA254 and LEU255, etc., as shown in Figure 4a,b.The most active derivative **4l** interacts with the active site ASN258 residue forming a hydrogen bond with phenol ring, and hydrogen atom interactions with a distance of 2.00 Å. The (N-H) hydrogen atom of the indole ring interacts with amino acid residues LYS352 and ALA317 forming hydrogen bond interactions with a distance of 2.80 and 1.86 Å respectively. The hydrophobic amino acids ALA316, ALA317, VAL 318, LEU248, LEU255 and MET259 interact with the aryl ring π-electrons and alkyl groups to form π-alkyl and π-σ interactions as shown in Figure 4a.

The second most active phosphonate derivative **4f** (5.2831) formed hydrogen bond interactions between the hydroxyl group oxygens of the phenyl ring and the hydrophobic amino acid LEU252 with a distance of 1.92 Å, whereas amino acid VAL315 interacts with the indole ring (-N-H) at a distance of 1.93 Å. Hydrophobic active site amino acid residues like LEU248, MET259, ALA250, LEU295 and the charged amino acid active site residues LYS254 and LYS352 interact with the aryl ring π- electrons and alkyl groups to form π-alkyl and π-σ interactions as shown in Figure 4b.

### 2.4. In Silico ADMET Predictions

At the beginning of the drug discovery and development process, prediction of drug-like properties of lead compounds is an imperative task as it is key step towards the success of the lead compounds. It has been observed that most active agents that show good biological activity but fail in clinical trials it is because of their poor drug like properties. The drug-like properties have been estimated by analyzing the absorption and distribution properties. We calculated and analyzed a range of physical descriptors and pharmaceutical relevant properties for ADMET prediction by using FAF Drugs 2 [53], and the data are as shown in Table 5. All the novel synthesized compounds exhibited noteworthy values for the various parameters analyzed and exhibited good drug-like characteristics based on Lipinski’s rule of five and its variants that characterized that these agents are likely to be orally active. The data obtained for all the synthesized methyl phosphonate derivatives **4**(**a**–**n**) are within the range of accepted values. None of the synthesized compounds violated the Lipinski’s rule [54,55] of five. The value of polar surface area (PSA), Log P and H/C ratio of synthesized compounds **4**(**a**–**n**) indicated good oral bioavailability. The parameters, like number of rotatable bonds and number of rigid bonds are linked with the intestinal absorption. All the synthesized compounds **4**(**a**–**n**) have shown good intestinal absorption. All the novel synthesized compounds **4**(**a**–**n**) were established to be non-toxic in nature. In silico assessment of all the novel synthetic compounds has shown that they have very good pharmacokinetic properties which is reflected in their physicochemical values and which is ultimately contributing for pharmacological properties of these molecules. By using a FAF Drugs 2 it was predicted that the compounds exhibited good % absorption (ABS) ranging from 66.82 to 76.98%, as shown in Table 5.

### 2.5. In Vivo Acute Oral Toxicity Study and Gross Behavioral Studies

Swiss albino mice treated with the newly synthesized compounds diethyl(4-chlorophenyl)(2-(2-oxoindolin-3-ylidene)hydrazinyl)methylphosphonate (**4b**) and (*Z*)-diethyl(3-ethoxy-4-hydroxy-phenyl)(2-(2-oxoindolin-3-ylidene)hydrazinyl)methylphosphonate (**4h**) were found to be free of any toxicity as per the acceptable range given by the OECD Guideline no. 425. No mortality was observed in mice when treated with the newly synthesized compounds **4b** and **4h** up to 2000 mg/kg. The results of the in vivo studies indicates that the lethal dose of the compounds **4b** and **4h** is above 2000 mg/kg body weight in mice, which points out that the compounds **4b** and **4h** can be considered to be safe and could be further developed in the coming years as potential anticancer agents. The data of the in vivo acute oral toxicity study and gross behavioral studies of the newly synthesized compounds **4b** and **4h** are summarized in Table 6.

## 3. Materials and Methods

### 3.1. General

All the chemicals used for synthesis were obtained from Merck (Darmstadt, Germany), Sigma (Mumbai, Maharashtra, India), Qualigens Fine chemicals (Mumbai, Maharashtra, India) and Himedia (Mumbai, Maharashtra, India).

### 3.2. Instrumentation

The FTIR spectra were obtained by means of a FTIR-4000 instrument (JASCO, Tokyo, Japan) and peaks were given in terms of wave number (cm^−1^). The ^1^H-NMR and ^13^C-NMR spectra of the synthesized compounds were recorded on an Avance II 400 NMR spectrometer (Bruker, Biospin AG Industriestrasse 26, CH-8117, Fallanden, Switzerland)at 400/100 MHz frequency in CDCl_3_ and using TMS as internal standard (chemical shift δ in ppm). The ^31^P-NMR spectra of compounds were recorded in CDCl_3_ using phosphoric acid (H_3_PO_4_) as external standard (chemical shift δ in ppm). The mass spectra were executed on a Micromass Q-Tof system (Waters, UK). Elemental analyses were done with a FLASHEA 112 analyzer (Shimadzu, Mumbai, Maharashtra, India) and all analyses were consistent (within 0.4%) with theoretical values. A Vibra Cell VCX-500 ultrasound synthesizer (Sonics, Newtown, CT, USA) equipped with a solid probe was employed for the synthesis of intermediate **1**. In vitro anti-cancer activity screening of the synthesized compounds was accomplished at the Anti-Cancer Drug screening facility (ACDSF) at ACTREC (Tata Memorial Centre, Navi Mumbai, India).

### 3.3. Synthesis

#### 3.3.1. Synthesis of 3-Hydrazonoindolin-2-one (**1**)

(A) Conventional method [56]

A mixture of indole-2,3-dione (isatin, 0.01 mol) and hydrazine hydrate (0.01 mol) in methanol (15 mL) was refluxed for 3–4 h in the presence of molecular sieves. The separated crystals were filtered, washed with a little amount of ethanol, dried and recrystallized from chloroform. The melting point of the synthesized compound **1** was found to be 284 °C, Yield 82%.

(B) Ultrasonication Method

Equimolar quantities of indole-2,3-dione (isatin, 0.01 mmol) and hydrazine hydrate (0.01 mmol) in the presence of glacial acetic acid (0.02 mmol) in absolute ethanol (5 mL) was sonicated by keeping the reaction mixture in an acoustic box containing an ultrasonic solid probe at 25–40 °C and at 25 amplitude for 15–20 min. The reaction mixture was concentrated and cooled. The obtained solid was filtered and dried. Recrystalization of the synthesized compounds was done using ethanol. Yield: 95%; melting point: 279–284 °C (uncorrected). Ultrasound method is thus seen to be better than the conventional method because it gives a better yield in 15–20 min against 3–4 h required for the conventional method. Also the amount of solvent required is less than that required for conventional method.

#### 3.3.2. General Procedure for the Synthesis of Diethyl(substituted phenyl/heteroaryl)(2-(2-oxoindolin-3-ylidene)hydrazinyl)methylphosphonates

Equimolar quantities of 3-hydrazonoindolin-2-one (**1**, 1 mmol), a substituted aromatic aldehyde/heteroaldehyde **2**(**a**–**n**) (1 mmol) and triethylphosphite (**3**, 1 mmol) were stirred at room temperature in absolute ethanol, in the presence of ceric ammonium nitrate (CAN, 0.003 mmol) as a catalyst. The TLC method was used to verify the completion of the reaction. After completion of the reaction, the reaction mixture was cooled and poured into water, filtered and the solid obtained was dried and recrystallized using ethanol. The time required for completion of reaction varies from 70 min to 89 min. The details are shown in Table 1. Our work represents a one pot Kabachnik-Fields synthesis of diethyl (substitutedphenyl/heteroaryl)(2-(2-oxoindolin-3-ylidene)hydrazinyl)-methylphosphonate derivatives from 3-hydrazonoindolin-2-one and substituted aromatic aldehyde/heteroaldehydes using CAN as a green catalyst at room temperature with better yield 84–95%.

*Diethyl(phenyl)(2-(2-oxoindolin-3-ylidene)hydrazinyl)methylphosphonate* (**4a**): Yield: 90%; M.P. 195–196 °C; ^1^H-NMR (CDCl_3_) δ: 1.2 (t, *J* = 7.11 Hz, 6H, 2×OCH_2_CH_3_),3.17 (d, *J* = 8.51 Hz, 1H, -CH), 4.70 (q, *J* = 7.11 Hz, 4H, 2×OCH_2_CH_3_), 7.10 (m, 9H, -CH), 8.61 (s, 1H, -NH), 10.90 (s, 1H, -NH of indole); ^13^C-NMR (CDCl_3_) δ: 16.31, 60.11, 63.32, 110.32, 119.25, 124.32, 126.25, 126.52, 128.12, 128.32, 129.22, 130.32, 162.11; ^31^P-NMR (CDCl_3_) δ: 19.90; ESI-MS: *m*/*z* calculated for C_19_H_22_N_3_O_4_P (M + H^+^): 388.84; found: 389.88 (M+1); IR (KBr) cm^−1^: 3340.31 (N-H stretching), 2960.41 (CH stretching of aromatic), 2837.21 (CH stretching of alkyl), 2300.23 (N-H stretching), 1620.33 (C=O stretching of amide), 1466.55 (CH Bending of CH_2_); Elemental analysis calculated for C_19_H_22_N_3_O_4_P: C, 58.91; H, 5.72; N, 10.85; P, 7.58; found; C, 58.88; H, 5.75; N, 10.87; P, 7.60.

*Diethyl(4-chlorophenyl)(2-(2-oxoindolin-3-ylidene)hydrazinyl)methylphosphonate* (**4b**): Yield 92%; M.P. 150–152 °C; ^1^H-NMR (CDCl_3_) δ: 1.20 (t, *J* = 7.11 Hz, 6H, 2×OCH_2_CH_3_), 2.53 (d, *J* = 8.11 Hz, 1H, -CH), 4.10 (q, *J* = 7.11 Hz, 4H, 2×OCH_2_CH_3_), 7.40 (m, 8H, -CH), 8.58 (s, 1H, -NH), 11.55 (s, 1H, -NH of indole); ^13^C-NMR (CDCl_3_) δ: 16.15, 40.17, 60.25, 78.07, 110.26, 117.96,127.79, 128.21, 128.83, 129.54, 130.19, 158.78, 164.52; ^31^P-NMR (CDCl_3_) δ: 18.84; ESI-MS: *m*/*z* calculated for C_19_H_21_ClN_3_O_4_P (M+1): 421.09; found: 422.33 (M+1); IR (KBr) cm^−1^: 3350.41 (N-H stretching), 2970.06 (CH stretching of aromatic), 2800.22 (CH stretching of alkyl), 2350.36 (N-H stretching), 1710.01 (C=O stretching of amide), 1454.75 (CH Bending of CH_2_); Elemental analysis calculated for C_19_H_21_ClN_3_O_4_P: C, 54.10; H, 5.02; N, 9.96; P, 7.34; found; C, 54.12; H, 5.04; N, 9.99; P, 7.39

*Diethyl(4-flurophenyl)(2-(2-oxoindolin-3-ylidene)hydrazinyl)methylphosphonate* (**4c**): Yield 95%; M.P. 176–180 °C; ^1^H-NMR (CDCl_3_) δ: 1.31 (t, *J* = 7.11 Hz, 6H, 2×OCH_2_CH_3_), 3.64 (d, *J*=8.45 Hz, 1H, -CH), 4.41 (q, *J* = 7.11 Hz, 4H, 2×OCH_2_CH_3_), 8.79 (m, 8H, -CH), 8.84 (s, 1H, -NH), 10.14 (s, 1H, -NH of indole); ^13^C-NMR (CDCl_3_) δ: 18.12, 65.21, 68.21, 123.32, 114.21, 117.14, 120.85, 127.55, 128.85, 131.36, 144.74, 146.96, 161.11, 164.85; ^31^P-NMR (CDCl_3_) δ: 18.54; ESI-MS: *m*/*z* calculated for C_19_H_21_FN_3_O_4_P (M + H^+^): 406.13; found: 407.20 (M + H^+^); IR (KBr) cm^−1^:3340.11 (N-H stretching), 2910.16 (CH stretching of aromatic), 2800.48 (CH stretching of alkyl), 2200.50 (N-H stretching), 1620.17 (C=O stretching of amide), 1464.47 (CH Bending of CH_2_); Elemental analysis calculated for C_19_H_21_FN_3_O_4_P: C, 56.30; H, 5.22; N, 10.37; P, 7.64 found; C, 56.33; H, 5.23; N, 10.40; P, 7.67

*(Z)-Diethyl(4-methoxyphenyl)(2-(2-oxoindolin-3-ylidene)hydrazinyl)methylphosphonate* (**4d**): Yield 89%; M.P. 178–179 °C; ^1^H-NMR (CDCl_3_) δ: 1.25 (t, *J* = 7.11 Hz, 6H, 2×OCH_2_CH_3_), 2.54 (s, 3H, O CH_3_), 3.42 (d, *J* = 8.41 Hz, 1H, -CH), 4.11 (q, *J* = 7.11 Hz, 4H, 2×OCH_2_CH_3_), 7.00 (m, 8H, -CH), 8.60 (s, 1H, -NH), 10.94 (s, 1H, -NH of indole); ^13^C-NMR (CDCl_3_) δ: 14.23, 55.13, 78.34, 79.88, 99.49, 110.93, 113.77, 119.25, 122.98, 126.47, 128.23, 129.85, 133.99, 144.85, 160.22, 168.98;^31^P-NMR (CDCl_3_) δ: 19.84; ESI-MS: *m*/*z* calculated for C_20_H_24_N_3_O_5_P (M + H^+^): 417.15; found: 418.42 (M + H^+^); IR (KBr) cm^−1^: 3350.11 (N-H stretching), 3070.76 (CH stretching of aromatic), 2800.96 (CH stretching of alkyl), 2300.11 (N-H stretching), 1610.47 (C=O stretching of amide), 1025.74 (-O- stretching); Elemental analysis calculated for C_20_H_24_N_3_O_5_P: C, 57.55; H, 5.80; N, 10.07; P, 7.42 found; C, 57.58; H, 5.82; N, 10.10; P, 7.45.

*Diethyl(3,4-dimethoxyphenyl)(2-(2-oxoindolin-3-ylidene)hydrazinyl)methylphosphonate* (**4e**): Yield 90%; M.P. 189–190 °C; ^1^H-NMR (CDCl_3_) δ: 1.21 (t, *J* = 7.11 Hz, 6H, 2×OCH_2_CH_3_), 2.55 (s, 6H, OCH_3_), 3.67 (d, *J* = 8.45 Hz, 1H, -CH), 4.21 (q, *J* = 7.11 Hz, 4H, 2×OCH_2_CH_3_), 7.37 (m, 7H, -CH), 8.42 (s, 1H, -NH), 10.03 (s, 1H, -NH of indole); ^13^C-NMR: (CDCl_3_) δ: 20.22, 58.69, 60.21, 61.12, 66.32, 111.78, 120.78, 121.36, 131.85, 132.11, 133.25, 141.74, 148.23, 150.41, 151.12, 167.47; ^31^P-NMR (CDCl_3_) δ: 18.94; ESI-MS: *m*/*z* calculated for C_21_H_26_N_3_O_6_P (M + H^+^): 448.16; found: 449.44 (M + H^+^); IR (KBr)cm^−1^: 3250.01 (N-H stretching), 2890.76 (CH stretching of aromatic), 2800.57 (CH stretching of alkyl), 2350.78 (N-H stretching), 1650.23 (C=O stretching of amide), 1002.44 (-O- stretching); Elemental analysis calculated for C_21_H_26_N_3_O_6_P: C, 56.37; H, 5.86; N, 9.39; P, 6.92 found; C, 56.40; H, 5.89; N, 9.41; P, 6.94.

*Diethyl(4-hydroxyphenyl)(2-(2-oxoindolin-3-ylidene)hydrazinyl)methylphosphonate* (**4f**): Yield 88%; M.P. 140–142 °C; ^1^H-NMR: (CDCl_3_) δ: 1.31 (t, *J* = 7.11 Hz, 6H, 2×OCH_2_CH_3_), 3.71 (d, *J* = 8.41 Hz, 1H, -CH), 4.54 (q, *J* = 7.11 Hz, 4H, 2×OCH_2_CH_3_), 5.61 (s, 1H, OH), 7.20 (m, 8H, -CH), 8.53 (s, 1H, -NH), 10.44 (s, 1H, -NH of indole); ^13^C-NMR (, CDCl_3_) δ: 17.26, 61.25, 69.74, 115.47,116.23, 117.63, 123.52, 129.85, 130.47, 134.52, 136.12, 145.32, 156.54, 164.41; ^31^P-NMR (CDCl_3_) δ:19.64; ESI-MS: *m*/*z* calculated for C_19_H_22_N_3_O_5_P (M + H^+^): 404.13; found: 405.37 (M + H^+^); IR (KBr) cm^−1^: 3600.10 (aromatic OH), 3440.44 (N-H stretching), 2980.88 (CH stretching of aromatic), 2800.01 (CH stretching of alkyl), 2280.21 (N-H stretching), 1710.22 (C=O stretching of amide); Elemental analysis calculated for C_19_H_22_N_3_O_5_P: C, 56.57; H, 5.50; N, 10.42; P, 7.68 found; C, 56.60; H, 5.54; N, 10.44; P, 7.70.

*Diethyl(4-hydroxy-3-methoxyphenyl)(2-(2-oxoindolin-3-ylidene)hydrazinyl)methylphosphonates* (**4g**): Yield: 94%; M.P. 112–114 °C; ^1^H-NMR (CDCl_3_) δ: 1.37 (t, *J* = 7.11 Hz, 6H, 2×OCH_2_CH_3_), 2.58 (s, 3H, OCH_3_), 3.36 (d, *J* = 8.41 Hz, 1H, CH), 4.14 (q, *J* = 7.11 Hz, 4H, 2×OCH_2_CH_3_), 6.84 (s, 1H, OH), 7.73 (m, 7H, CH), 9.77 (s, 1H, -NH), 10.55 (s, 1H, -NH of indole); ^13^C-NMR (CDCl_3_) δ: 16.05, 40.17, 55.40, 62.57, 110.36, 116.74, 120.48, 125.29, 132.66,134.25, 136.24, 138.59, 138.90, 144.46, 150.94, 190.22; ^31^P-NMR (CDCl_3_) δ: 19.94; ESI-MS: *m*/*z* calculated for C_20_H_24_N_3_O_6_P (M + H^+^): 434.14; found: 435.39 (M + H^+^); IR (KBr) cm^−1^:3610.45 (aromatic OH), 3450.64 (N-H stretching), 2996.74 (CH stretching of aromatic), 2830.74 (CH stretching of alkyl), 2310.21 (N-H stretching), 1680.12 (C=O stretching of amide), 1030.14 (-O-stretching); Elemental analysis calculated for C_20_H_24_N_3_O_6_P: C, 55.43; H, 5.51; N, 9.70; found; C, 55.49; H, 5.60; N, 9.75.

*(Z)-Diethyl(3-ethoxy-4-hydroxyphenyl)(2-(2-oxoindolin-3-ylidene)hydrazinyl)methylphosphonate* (**4h**): Yield 92%; M.P. 160–162 °C; ^1^H-NMR (CDCl_3_) δ: 1.40 (t, *J* = 7.11 Hz, 9H, 3×OCH_2_CH_3_), 3.90 (d, *J* = 8.41 Hz, 1H, CH), 4.51 (q, *J* = 7.11 Hz, 6H, 3×OCH_2_CH_3_), 5.42 (s, 1H, OH), 7.71 (m, 7H, CH), 8.8 (s, 1H, -NH),10.72 (s, 1H, -NH of indole); ^13^C-NMR (CDCl_3_) δ: 16.21, 16.52,18.21, 61.52, 64.85, 68.74, 117.12, 116.41, 119.32, 121.92, 122.74, 130.96, 133.12, 136.24, 141.23, 142.74, 167.74; ^31^P-NMR (CDCl_3_) δ: 18.65; ESI-MS: *m*/*z* calculated for C_21_H_26_N_3_O_6_P (M + H^+^): 448.16; found: 449.40 (M + H^+^); IR (KBr) cm^−1^: 3550.50 (-OH),3420.32 (N-H stretching), 2999.45 (CH stretching of aromatic), 2813.87 (CH stretching of alkyl), 2350.52 (N-H stretching), 1710.72 (C=O stretching of amide), 1020.42 (-O- stretching); Elemental analysis calculated for C_21_H_26_N_3_O_6_P: C, 56.37; H, 5.86; N, 9.39; P, 6.92 found; C, 56.40; H, 5.88; N, 9.41; P, 6.94.

*(Z)-Diethyl(2-(2-oxoindolin-3-ylidene)hydrazinyl)(thiophen-2-yl)methylphosphonate* (**4i**): Yield 87%; M.P. 179–182 °C; ^1^H-NMR (CDCl_3_) δ: 1.01 (t, *J* = 7.11 Hz, 6H, 2×OCH_2_CH_3_), 3.64 (d, *J* = 8.44 Hz, 1H, CH), 3.82 (s, 1H, CH), 4.51 (q, *J* = 7.11 Hz, 4H, 2×OCH_2_CH_3_), 7.81 (m, 6H, CH), 8.57 (s, 1H, -NH), 10.77 (s, 1H, -NH of indole); ^13^C-NMR: (CDCl_3_) δ: 17.12, 63.52, 69.96, 119.12, 120.35, 124.18, 128.47, 129.96, 130.18, 150.52, 152.74, 155.54, 165.65, 170.65,174.96; ^31^P-NMR (CDCl_3_) δ: 18.45; ESI-MS: *m*/*z* calculated for C_17_H_20_N_3_O_4_PS (M + H^+^): 394.09; found: 395.48 (M + H^+^);IR (KBr) cm^−1^: 3520.72 (N-H stretching), 2912.88 (CH stretching of aromatic), 2800.32 (CH stretching of alkyl), 2340.54 (N-H stretching), 1710.72 (C=O stretching of amide); Elemental analysis calculated for C_17_H_20_N_3_O_4_PS: C, 51.90; H, 5.12; N, 10.68; P, 7.87; S, 8.15 found; C, 51.91; H, 5.14; N, 10.70; P, 7.89; S, 8.17.

*(Z)-Diethylfuran-2-yl(2-(2-oxoindolin-3-ylidene)hydrazinyl)methylphosphonate* (**4j**): Yield 84%; M.P. 176–178 °C; ^1^H-NMR (CDCl_3_) δ: 1.20 (t, *J*=7.11 Hz, 6H, 2×OCH_2_CH_3_), 3.81 (s, 1H, CH), 3.42 (d, *J* = 8.44 Hz, 1H, CH), 4.62 (q, *J* = 7.11 Hz, 4H, 2×OCH_2_CH_3_), 7.98 (m, 6H, CH), 8.44 (s, 1H, -NH), 10.45 (s, 1H, -NH of indole); ^13^C-NMR (CDCl_3_) δ: 22.21, 50.11, 65.52, 112.31, 119.95, 121.36, 128.63, 130.36, 131.21, 133.65, 147.33, 151.25, 153.23, 155.39, 164.21; ^31^P-NMR (CDCl_3_) δ: 18.56; ESI-MS: *m*/*z* calculated for C_17_H_20_N_3_O_5_P (M + H^+^): 378.12; found: 379.33 (M + H^+^);IR (KBr)cm^−1^: 3280.32 (NH stretching), 2945.46 (CH aromatic), 2850.63 (CH stretching of alkyl), 2440.85 (C=N stretching), 1710.33 (C=O stretching), 1070.10 (-O- stretching); Elemental analysis calculated for C_17_H_20_N_3_O_5_P: C, 54.11; H, 5.34; N, 11.14; P, 8.21 found; C, 54.14; H, 5.36; N, 11.16; P, 8.24.

*(Z)-Diethyl (2-hydroxyphenyl)(2-(2-oxoindolin-3-ylidene)hydrazinyl)methylphosphonate* (**4k**): Yield 88%; M.P.152–154 °C; ^1^H-NMR (DMSO-d_6_) δ: 1.29 (t, *J* = 7.11 Hz, 6H, 2×OCH_2_CH_3_), 4.07 (q, *J* = 7.12 Hz, 4H, 2×OCH_2_CH_3_),5. 35 (s, 1H, OH), 6.80–8.08 (m, 9H, aromatic), 8.0 (s, 1H, -NH), 10.46 (s, 1H, -NH of indole); ^13^C-NMR: (DMSO-d_6_) δ ppm: 16.31, 61.33,62.24, 115.41, 117.45, 119.05, 121.33, 121.56, 124.42, 128.46, 128.73, 129.11, 131.91, 133.22, 134.59, 141.44, 155.81, 168.44; ^31^P-NMR (CDCl_3_) δ: 19.56; ESI-MS: *m*/*z* calculated for C_19_H_22_N_3_O_5_P (M + H^+^): 403.13; found: 404.37 (M + H^+^); IR (KBr) cm^−1^: NH 3313.45 (N-H stretching), 3033.42 (C-H stretching of aromatic), 2653.64 (C-H stretching of alkyl), 2133.63 (C=N Stretching), 1723.05 (C-O stretching), 1690.80 (C=O stretching), 1554.65 (C-N Stretching), 1034.65 (O- stretching),Elemental Analysis calculated for C_19_H_22_N_3_O_5_P: C, 56.57; H, 5.50; N, 10.42; P, 7.68 found; C, 56.59; H, 5.52; N, 10.44; P, 7.69.

*(Z)-Diethyl (4-hydroxy-3-methylphenyl)(2-(2-oxoindolin-3-ylidene)hydrazinyl)methylphosphonate* (**4l**): Yield 75%; M.P.190–192 °C; ^1^H-NMR (DMSO-d_6_) δ: 1.29 (t, *J* = 7.11 Hz, 6H, 2×OCH_2_CH_3_), 2.15 (s, 1H, CH_3_), 4.07.(q, *J* = 7.12 Hz, 4H, 2×OCH_2_CH_3_), 5. 35 (s, 1H, OH), 6.80–8.08 (m, 9H, aromatic), 8.0 (s, 1H, -NH), 10.45 (s, 1H, -NH of indole); ^13^C-NMR: (DMSO-d_6_) δ ppm: 15.32, 16.33, 62.22, 67.72, 115.4, 118.44, 119.93, 124.49, 124.88, 125.94, 128.32, 129.81, 130.59, 131.72, 133.49, 141.45, 152.21, 168.41;^31^P-NMR (CDCl_3_) δ: 19.56; ESI-MS: *m*/*z* calculated for C_20_H_24_N_3_O_5_P (M + H^+^): 417.15; found: 417.40; IR (KBr) cm^−1^: NH 3303.34 (N-H stretching), 3013.32 (C-H stretching of aromatic), 2653.64 (C-H stretching of alkyl), 2133.63 (C=N Stretching), 1723.05 (C-O stretching), 1690.80 (C=O stretching), 1554.65 (C-N Stretching), 1034.65 (O- stretching),Elemental Analysis calculated for C_20_H_24_N_3_O_5_P: C, 57.55; H, 5.80; N, 10.07; P, 7.42 found; C, 57.59; H, 5.82; N, 10.09; P, 7.43.

*(Z)-Diethyl (4-nitrophenyl)(2-(2-oxoindolin-3-ylidene)hydrazinyl)methylphosphonate* (**4m**): Yield 90%; M.P.172–174 °C; ^1^H-NMR (400 MHz, DMSO-d_6_) δ: 1.29 (t, *J* = 7.11 Hz, 6H, 2×OCH_2_CH_3_), 4.07 (q, *J* = 7.11 Hz, 4H, 2×OCH_2_CH_3_), 5. 35 (s, 1H, OH), 6.80–8.08 (m, 9H, aromatic), 8.0 (s, 1H, -NH), 10.45 (s, 1H, -NH of indole);^13^C-NMR: (DMSO-d_6_) δ ppm: 16.37, 62.24, 68.88, 117.76, 119,82, 123.37, 124.43, 127.94, 129.53, 131.22, 134.54, 141.14, 142.39, 145.45, 168.76; ^31^P-NMR (,CDCl_3_) δ: 19.56; ESI-MS: *m*/*z* calculated for C_19_H_21_N_4_O_6_P: (M + H^+^): 432.12, found: 433.37; IR (KBr) cm^−1^: NH 3303.34 (N-H stretching), 3013.32 (C-H stretching of aromatic), 2653.64 (C-H stretching of alkyl), 2133.63 (C=N Stretching), 1723.05 (C-O stretching), 1690.80 (C=O stretching), 1554.65 (C-N Stretching), 1034.65 (O- stretching); Elemental Analysis calculated for C_19_H_21_N_4_O_6_P: C, 52.78; H, 4.90; N, 12.96; P, 7.16 found; C, 52.79; H, 4.93; N, 12.98; P, 7.17.

*(Z)-Diethyl (4-methylthiazole-5-yl)(2-(2-oxoindolin-3-ylidene)hydrazinyl)methylphosphonate*(**4n**): Yield 70%; M.P. 188–190 °C; ^1^H-NMR (DMSO-d_6_) δ: 1.29 (t, *J* = 7.11 Hz, 6H, 2×OCH_2_CH_3_), 2.46 (s, 1H, CH_3_), 4.07.(q, *J* = 7.12 Hz, 4H, 2×OCH_2_CH_3_), 5. 35 (s, 1H, OH), 6.80–8.08 (m, 9H, aromatic), 8.0 (s, 1H, -NH), 10.45 (s, 1H, -NH of indole); ^13^C-NMR: (DMSO-d_6_) δ ppm: 14.73, 16.39, 62.28, 65.77, 117.78, 121.3, 124.48, 129.47, 130.13, 131.1 (C), 133,34, 141.22, 148.77, 151.34, 168.79; ^31^P-NMR (,CDCl_3_) δ: 19.54; ESI-MS: *m*/*z* calculated for C_17_H_21_N_4_O_4_PS: (M + H^+^): 408.10, found: 408.41; IR (KBr) cm^−1^: NH 3303.34 (N-H stretching), 3013.32 (C-H stretching of aromatic), 2653.64 (C-H stretching of alkyl), 2133.63 (C=N Stretching), 1723.05 (C-O stretching), 1690.80 (C=O stretching), 1554.65 (C-N Stretching), 1034.65 (O-stretching),2723.65 (COOH); Elemental Analysis calculated for C_17_H_21_N_4_O_4_PS: C, 47.57; H, 5.10; N, 12.33; P, 6.82 found; C, 47.59; H, 5.13; N, 12.38; P, 6.80.

### 3.4. In Vitro Anticancer Activity

All the newly synthesized compounds were screened for their in vitro anticancer activity against six cancer cell lines: SK-MEL-2, MCF-7, IMR-32, MG-63, HT-29 and Hep-G2 by SRB assay, using adriamycin as a standard drug [57]. All the synthesized derivatives **4**(**a**–**n**) were also tested for their cytotoxic effect on normal cell lines i.e., NIH/3T3 (murine embryonic fibroblast by the SRB assay method. The cell lines were grown in RPMI 1640 medium containing 10% fetal bovine serum and 2 mM l-glutamine. For the present screening experiments, cells were inoculated into 96 well microtiter plates in 90 µL at 5000 cells per well. After cell inoculation, the microtiter plates were incubated at 37 °C, 5% CO_2_, 95% air and 100% relative humidity for 24 h prior to addition of experimental drugs. Experimental drugs were solubilized in appropriate solvent to prepare stock of 10^−2^ concentration. At the time of experiment four 10-fold serial dilutions were made using complete medium. Aliquots of 10 µL of these different drug dilutions were added to the appropriate micro-titer wells already containing 90 µL of medium, resulting in the required final drug concentrations. After compound addition, plates were incubated at standard conditions for 48 h. and assay was terminated by the addition of cold trichloroacetic acid (TCA). Cells were fixed in situ by the gentle addition of 50 µL of cold 30% (*w*/*v*) TCA (final concentration, 10% TCA) and incubated for 60 min at 4 °C. The supernatant was discarded; the plates were washed five times with tap water and air dried.

Sulforhodamine B (SRB) solution (50 µL) at 0.4% (*w*/*v*) in 1% acetic acid was added to each of the wells, and plates were incubated for 20 min at room temperature. After staining, unbound dye was recovered and the residual dye was removed by washing five times with 1% acetic acid. The plates were air dried. Bound stain was subsequently eluted with 10 mM trizma base, and the absorbance was read on an ELISA plate reader at a wavelength of 540 nm with 690 nm reference wavelength. All the tests were repeated in at least three independent experiments at the concentrations of 10, 20, 40 and 80 µg/mL [57].

### 3.5. Docking Study

The computational study i.e., molecular docking study, was started by sketching the2D form of the structures of all synthesized compounds using the sketch modules of SYBYL-X 2.1.1. 2D [58] formss of the compounds then subjected to the ligand library preparation module by keeping the preparation protocol as surface for searching where it generates a single lowest strain energy tautomer/stereoisomer and all necessary structural properties were added and finally a 3D prepared conformation of each compounds was stored in SYBYL-Mol2 file format. To perform molecular docking a three dimensional X-ray crystal structure of tubulin (PDB ID: 1SA0 Resolution 3.58 Å) [59] complex with colchicine was used. Many TRK inhibitors (TRKs) have been produced and tested in the clinic by now. The crystal structures of c-kit receptor protein-tyrosine kinase in complex with STI-571 (imatinib or Gleevec) were picked from the Protein Data Bank (PDB) (http://www.rcsb.org/pdb/explore/explore.dostructureId=1T46) (PDB code: 1t46). The synthesized compounds **4**(**a**–**n**) were subjected to a molecular docking study performed with the Surflex-Dock module of the Sybyl2.1.1 package following standard procedures for understanding the binding interactions with human TRKs1 enzyme (PDB ID: 1t46). All the synthesized compounds showed very good binding interactions in the active site of the selected receptors. The most active compounds from the synthesized series which have exhibited very high docking scores value against selected receptors and had a good binding affinity predicated by non-covalent interactions such as hydrogen bond interaction, VDW interaction, carbon, hydrogen bond interaction, π-Anion interaction, π-π shaped interaction, alkyl interaction, π-σ and π-alkyl interactions. To represent the details of docking score the following terms is used as total score: crash score: as the degree of inappropriate penetration by the ligand into the protein and of interpenetration between ligand atoms that are separated by rotatable bonds of compounds and polar-score which gives an idea about the contribution of the polar non-hydrogen bonding interactions to the total score, as shown in Table 3 and Table 4.

### 3.6. In-Silico Bioavailability Predictions

The bioavailability properties were predicted and it was seen that the compounds displayed an admirable % ABS (66.82–76.98%, shown in Table 5). Absorption (% ABS) was calculated by: % ABS = 109−(0.345 X TPSA). In the current research study, molecular volume (MV), molecular weight (MW), logarithm of partition coefficient (miLog P), number of hydrogen bond acceptors (n-ON), number of hydrogen bonds donors (n-OHNH), topological polar surface area (TPSA), number of rotatable bonds (n-ROTB), number of rigid bonds (Rig Bond), Rings, ratio H/C and Lipinski’s rule of five were calculated using FAF Drugs 2. None of the synthesized compounds violated the Lipinski’s rule of five or its variants. All the synthesized compounds **4**(**a**–**n**) were found to be non-toxic, as predicted by using FAF Drugs 2.

### 3.7. In Vivo Acute Oral Toxicity Study and Gross Behavioral Studies

The in vivo acute oral toxicity study for the newly synthesized scaffolds **4b** and **4h** was performed according to the OECD Guideline no. 425 [60] using Swiss albino mice (18–22 gm weight) quarantined at the animal house at Y.B. Chavan College of Pharmacy, Aurangabad IAEC approval number CPCSEA/IAEC/P’col-52/2015-16/115. Each group consisted of six mice (overnight fasted) and kept in a colony cage at 25 ± 2 °C with 55% relative humidity and 12 h of light and dark cycle. A specified dose of 100, 250, 500, 750, 1000, 1500 and 2000 mg/kg body weight of mice was administered orally as a single dose. The acute toxic symptoms and the behavioral changes produced by the test compounds were observed continuously for 4 h periods at 8th, 12th and 24th h. Onset of toxic symptoms and the gross behavioral changes were also recorded. These animals were maintained for further 10 days with observations made daily. In case the animal appeared moribund (dying) the animal was sacrificed in a humane way and it is considered to have died because of toxicity.

## 4. Conclusions

Novel fourteen diethyl (substituted phenyl/heteroaryl)(2-(2-oxoindolin-3ylidene)hydrazinyl)-methylphosphonate derivatives **4**(**a**–**n**)were synthesized by a one pot reaction using CAN as a green catalyst. The compounds were characterized by TLC, IR, NMR, mass spectrometry and elemental analysis. The in-vitro anticancer activity was evaluated against six human cancer cell lines such as MCF-7, IMR-32, SK-MEL-2, MG-63, HT-29 and Hep-G2by the SRB assay method. Adriamycin was used as a positive control. All the synthesized derivatives have shown excellent in vitro anticancer activity against MG-63 cancer cell lines, equipotent to that of the standard drug adriamycin. The compound **4l** with a 4-hydroxy3-methyl group on the phenyl ring was also found to be equipotent to the standard drug adriamycin against the HT-29 and Hep-G2 cancer cell lines. The synthesized compounds **4**(**a**–**n**) were found to be selective towards cancer cells since they did not exhibit cytotoxicity on normal tissue cells even at GI_50_ > 250 μM. The treatment of selected cancer cell lines with the synthesized compounds showed apoptotis and morphological changes like cell shrinkage, cell wall deformation and reduced number of viable cells. A computational study i.e., a molecular docking study of the synthesized compounds **4**(**a**–**n**), was carried out to know the binding interactions of the synthesized derivatives with the tyrosine kinase receptor and microtubules. From the results of the molecular docking study, it was observed that the methyl phosphonate derivatives have dual inhibition potential, inhibiting human TRKS and microtubules.

The synthesized compounds can act as dual inhibitors, if developed as drug molecules in the future. They can serve as good anticancer agents against cancers for which resistance has developed. The synthesized compounds **4**(**a**–**n**) showed no cytotoxicity towards normal tissue cells. It’s very vital for cancer treatment that the anticancer drugs have the property of high efficiency and low toxicity. The synthesized compounds **4**(**a**–**n**) were identified to be selective towards cancer cells in view of the fact that they did not display cytotoxicity even at GI_50_ > 250 μM on normal tissue cells. In silico bioavailability studies indicated that compounds have a good in silico % absorption (66.82% to 76.98%). All the synthesized compounds **4**(**a**–**n**) were identified to be non-toxic in nature. All the above information suggests that the novel compounds of the current series can serve as a lead scaffold in the design, development and synthesis of new anticancer agents, especially for bone, liver and melanoma types of cancers.

## Figures and Tables

**Table 1:** Physical constant data for diethyl (substitutedphenyl/hetery)(2-(2-oxoindolin-3-ylidene) hydrazinyl)methylphosphonates **4**(**a**–**n**).

Code	R	Molecular Formula	Molecular Weight (gm)	Time Required (min)	% Yield	Melting Point (°C)
**4a**	Phenyl	C_19_H_22_N_3_O_4_P	387.37	75	90	195–196
**4b**	4-Chlorophenyl	C_19_H_21_ClN_3_O_4_P	421.81	70	92	150–152
**4c**	4-Fluorophenyl	C_19_H_21_FN_3_O_4_P	405.36	75	95	176–180
**4d**	4-Methoxyphenyl	C_20_H_24_N_3_O_5_P	417.40	85	89	178–179
**4e**	3,4-Dimethoxyphenyl	C_21_H_26_N_3_O_6_P	447.42	90	90	189–190
**4f**	4-Hydroxyphenyl	C_19_H_22_N_3_O_5_P	403.37	80	88	140–142
**4g**	4-Hydroxy-3-methoxyphenyl	C_20_H_24_N_3_O_6_P	433.39	75	94	112–114
**4h**	4-hyroxy-3-ethoxyphenyl	C_21_H_26_N_3_O_6_P	447.44	80	92	160–162
**4i**	Thiophen-2-yl	C_17_H_20_N_3_O_4_PS	393.40	80	87	179–182
**4j**	Furan-2-yl	C_17_H_20_N_3_O_5_P	377.33	80	84	176–178
**4k**	2-Hydroxyphenyl	C_19_H_22_N_3_O_5_P	403.37	75	88	152–154
**4l**	4-Hydroxy-3-methylphenyl	C_20_H_24_N_3_O_6_P	417.40	82	75	190–192
**4m**	4-Nitrophenyl	C_19_H_21_N_4_O_6_P	432.37	89	90	172–174
**4n**	4-Methylthiazole-5-yl	C_17_H _21_N_4_O_4_PS	408.10	75	70	188–190

**Table 2:** In vitro Anticancer activity data of the novel synthesized compounds **4**(**a**–**n**).

Compounds			GI_50 (_µM)					
	R	MCF-7	IMR-32	SKMEL-2	MG-63	HT-29	Hep-G2	NIH/3T3
**4a**	Phenyl	>100	>100	56.9	<0.1	56.9	55.2	>250
**4b**	4-Chlorophenyl	67.2	>100	41.4	<0.1	46.9	18.6	>250
**4c**	4-Fluorophenyl	>100	>100	57.5	<0.1	20.8	32.0	>250
**4d**	4-Methoxyphenyl	>100	>100	65.7	<0.1	<0.1	45.3	>250
**4e**	3,4-Dimethoxyphenyl	>100	>100	55.2	<0.1	<0.1	12.2	>250
**4f**	4-Hydroxyphenyl	>100	<0.1	51.6	<0.1	<0.1	12.2	>250
**4g**	4-Hydroxy-3-methoxyphenyl	>100	>100	24.0	<0.1	12.2	32.0	>250
**4h**	4-hyroxy-3-ethoxyphenyl	70.7	>100	51.0	<0.1	4.1	43.8	>250
**4i**	Thiophen-2-yl	>100	>100	55.5	<0.1	<0.1	<0.1	>250
**4j**	Furan -2-yl	>100	>100	93.9	<0.1	<0.1	44.6	>250
**4k**	2-Hydroxyphenyl	>100	44.1	66.2	<0.1	26.1	11.7	>250
**4l**	4-Hydroxy-3-methylphenyl	78.4	89.8	76.4	<0.1	<0.1	<0.1	>250
**4m**	4-Nitrophenyl	77.2	56.6	44.2	<0.1	<0.1	13.3	>250
**4n**	4-Methylthiazole-5-yl	58.2	67.8	33.1	<0.1	<0.1	24.9	>250
**^a^ADR**	-	<0.1	<0.1	<0.1	<0.1	<0.1	<0.1	82.2

^a^ ADR: Adriamycin used as standard drug. GI_50_ is the concentration exhibiting 50% inhibition of the growth, as compared to the growth of control. MCF-7: Human breast cancer cell line; IMR-32: Neuroblastoma cell line; SKMEL-2: Human melanoma cancer cell line; MG-63: Human osteosarcoma cell line; HT-29: Human colon cancer cell line; Hep-G2: Human hepatoma cell line; NIH/3T3: Normal murine embryonic fibroblast cell line.

**Table 3:** Molecular docking study data of the compounds **4**(**a**–**n**) in TRK.

Compound ID	Total Score −Log(ki)	Crash	Polar
**4a**	5.1189	−1.2354	1.997
**4b**	5.0299	−0.889	3.0602
**4c**	4.7873	−0.8462	1.6198
**4d**	5.4734	−1.1481	1.6983
**4e**	5.3336	−1.9652	3.1869
**4f**	6.0396	−0.7543	2.4298
**4g**	5.5786	−1.5587	3.8036
**4h**	4.8916	−2.8753	3.8417
**4i**	3.5991	−1.0332	3.0929
**4j**	4.5136	−0.6275	3.1716
**4k**	5.7714	−1.0431	1.5553
**4l**	4.9855	−0.6461	1.4008
**4m**	5.6219	−0.919	3.4772
**4n**	5.5030	−0.9370	3.7706

Total score: Total docking score: Crash: degree of inappropriate penetration by the ligand into the protein and of interpenetration between ligand atoms that are separated by rotatable bonds; Polar: contribution of the polar non-hydrogen bonding interactions to the total score.

**Table 4:** Molecular docking of the synthesized compounds **4**(**a**–**n**) in tubulin receptor.

Sr. No	Total Score (−Log Ki)	Crash Score	Ploar Score
**4a**	4.09	−1.14	1.01
**4b**	4.19	−2.77	1.65
**4c**	4.40	−1.56	0.78
**4d**	4.60	−1.22	1.15
**4e**	3.62	−2.25	1.46
**4f**	5.28	−1.08	2.45
**4g**	5.11	−1.01	3.01
**4h**	4.27	−1.38	1.82
**4i**	3.52	−1.53	2.10
**4j**	4.32	−1.34	1.99
**4k**	3.52	−2.35	1.29
**4l**	6.12	−1.15	2.11
**4m**	4.98	−0.93	1.67
**4n**	4.72	−2.08	1.48
ADR	3.77	−1.58	3.47

**Table 5:** Pharmacokinetic parameters of the synthesized compounds **4**(**a**–**n**) for good oral bioavailability.

Entry	% ABS	TPSA(A2)	n-ROTB	MV	MW	miLogP	n-ON	n-OHNH	Lipinski Violation	Toxicity
Rule	**-**	**-**	**-**	**-**	<500	<5	<10	<5	<1	**-**
**4a**	76.98	92.79	8	341.53	387.38	2.92	7	2	0	Non Toxic
**4b**	76.98	92.79	8	355.06	421.82	3.59	7	2	0	Non Toxic
**4c**	76.98	92.79	8	346.46	405.37	3.08	7	2	1	Non Toxic
**4d**	73.70	102.3	9	367.07	417.40	2.97	8	2	0	Non Toxic
**4e**	70.61	111.26	10	392.62	447.43	2.56	9	2	0	Non Toxic
**4f**	69.94	113.2	8	349.55	403.38	2.44	3	3	0	Non Toxic
**4g**	66.82	122.25	9	375.09	433.40	2.25	9	3	0	Non Toxic
**4h**	66.82	122.25	10	391.89	447.43	2.63	9	3	0	Non Toxic
**4i**	76.98	92.79	8	332.34	393.40	2.81	7	2	0	Non Toxic
**4j**	72.45	105.93	8	323.10	377.34	2.17	8	2	1	Non Toxic
**4k**	67.92	113.2	9	375.09	403.39	2.25	9	3	0	Non Toxic
**4l**	67.92	119.06	9	367.07	417.39	2.97	8	2	0	Non Toxic
**4m**	59.48	92.79	9	364.86	432.37	2.86	10	3	0	Non Toxic
**4n**	50.05	170.86	9	349.04	407.43	1.77	8	1	0	Non Toxic

% ABS—Percentage of absorption; TPSA—topological polar surface area; n-ROTB—number of rotatable bonds; MV—molecular volume; MW—molecular weight; miLogP—logarithm of partition coefficient; n-ON—number of hydrogen bond acceptors; n-OHNH—number of hydrogen bonds donors.

**Table 6:** In vivo acute oral toxicity study and gross behavioral studies of the compounds **4b** and **4h**.

			Additional Observation								Behavioral Observation										
Entry	Toxicity	No. of Death	Skin and Fur	Eyes Lacrimation	Salivation	Diarrhea	Respiration	Straub Tail	Pilo Erection	Convulsions	Motor Activity	Stereotypy	Tremors	Catalepsy	Sedation	Hypnosis	Writhing	Muscle Spasm	Analgesia	Arching & Rolling	Writhing
**4b**	Nil	Nil	N	N	N	N	N	N	Y	N	N	N	N	N	N	N	N	N	N	N	N
**4h**	Nil	Nil	N	N	N	N	N	N	Y	N	Y	N	N	N	N	N	N	N	N	N	N

N. no, Y: Yes.

## Supplementary Materials

The following are available online. Figures S1–S7.
