# Supplementary material for: New 2-Oxoindolin Phosphonates as Novel Agents to Treat Cancer: A Green Synthesis and Molecular Modeling
Source: Molecules. 2018 Aug 8;23(8):1981. doi: 10.3390/molecules23081981 (PMC6222476; doi:10.3390/molecules23081981)
Supplement: Supplementary file 1 [file molecules-23-01981-s001.pdf]

New 2-oxoindolin phosphonates As Novel Agents to Treat Cancer: A Green Synthesis and Molecular Modeling.

Shailee V. Tiwari [1], Nawaz S. Sharif [1], Rekha I. Gajare [1], Julio A. Seijas Vazquez[2], Jaiprakash N. Sangshetti[1], Manoj D. Damale[1], Anna Pratima G.Nikalje [1] *

[1]Y. B. Chavan College of Pharmacy, Dr. Rafiq Zakaria Campus, Rauza Baug, Aurangabad 431001, Maharashtra, India; shailee2010@gmail.com, nawajsharifsk@gmail.com

[2] Departamento de Química Orgánica, Facultad de Ciencias, Universidad of Santiago De Compostela, Alfonso X el Sabio, Lugo 27002, Spain; julioa.seijas@usc.es, pilar.vazquez.tato@usc.es

*Corresponding author Email: annapratimanikalje@gmail.com; contact: +91 9168929111

2. Results

2.1. Chemistry

The mechanism of synthesis is as shown in Figure S1

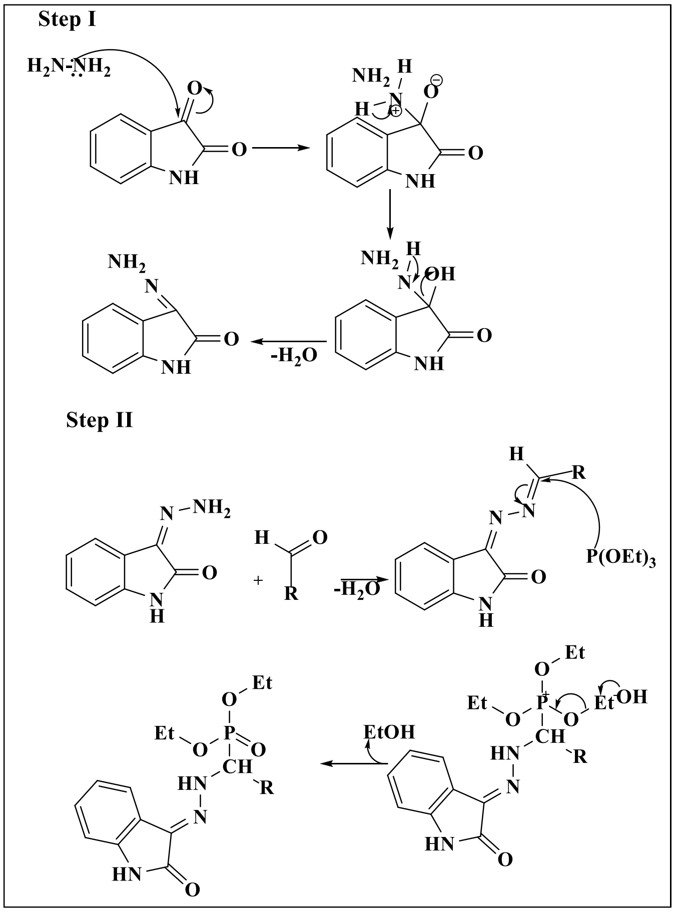

Figure S1. The proposed mechanism for the synthesis of **4(a-n)** derivatives.

In Vitro **Anticancer evaluation**

In vitro anticancer activity images which were captured under the Eclipse Ti-S Inverted Research Microscope-Nikon and the images were processed using NIS-Elements software. The images of the *in vitro* anticancer activity of all the synthesized compounds **4(a-n)** on the MCF-7, IMR-32, SK-MEL-2, MG-63, HT-29 and Hep-G2 cancer cell lines are as shown in Figure S2, Figure S3, Figure S4, Figure S5, Figure S6 and Figure S7, respectively.

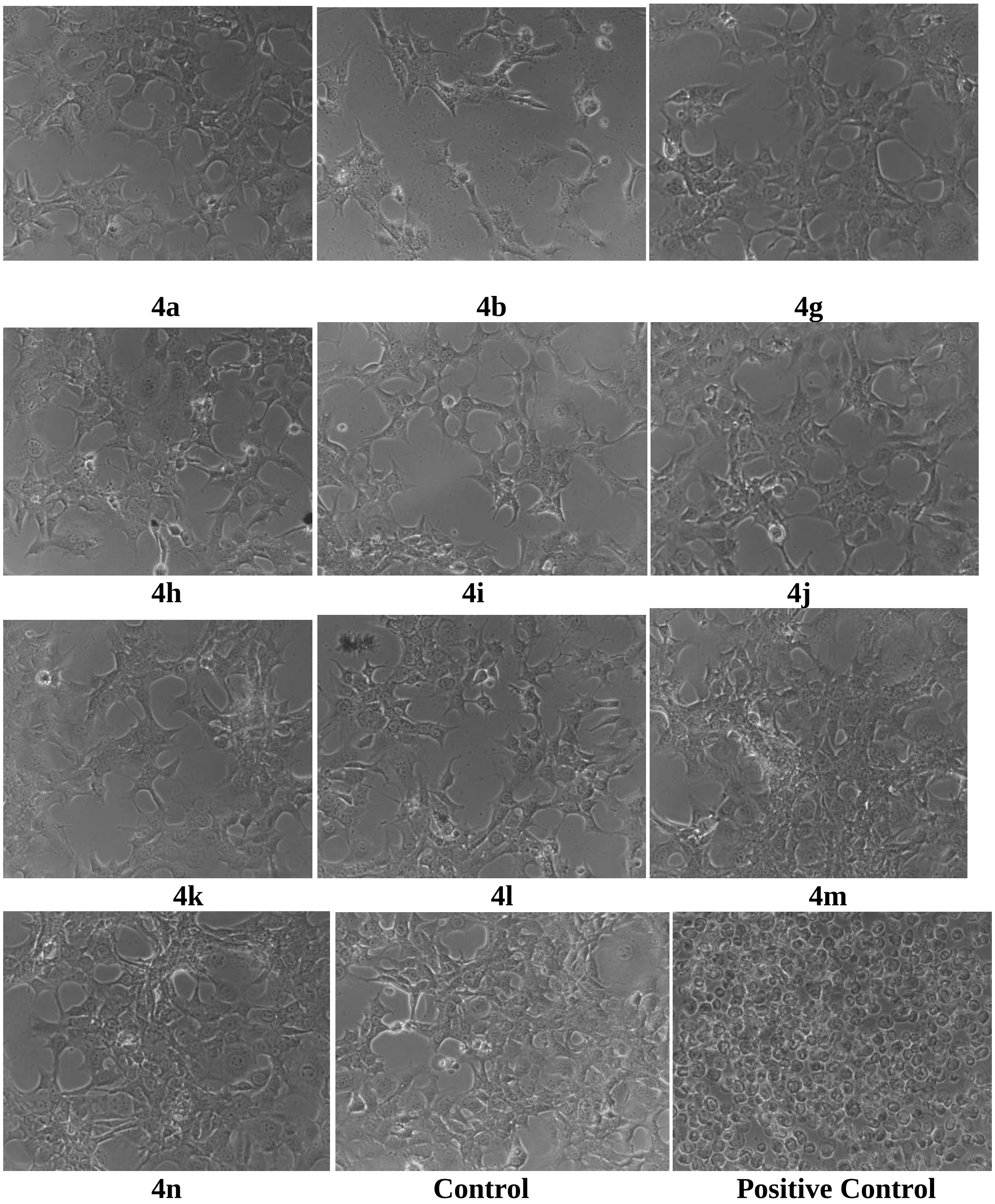

Figure S2. Images for *in-vitro* anticancer activity against MCF-7 cell line of the synthesized compounds **4 (a-n)**, control and positive control.

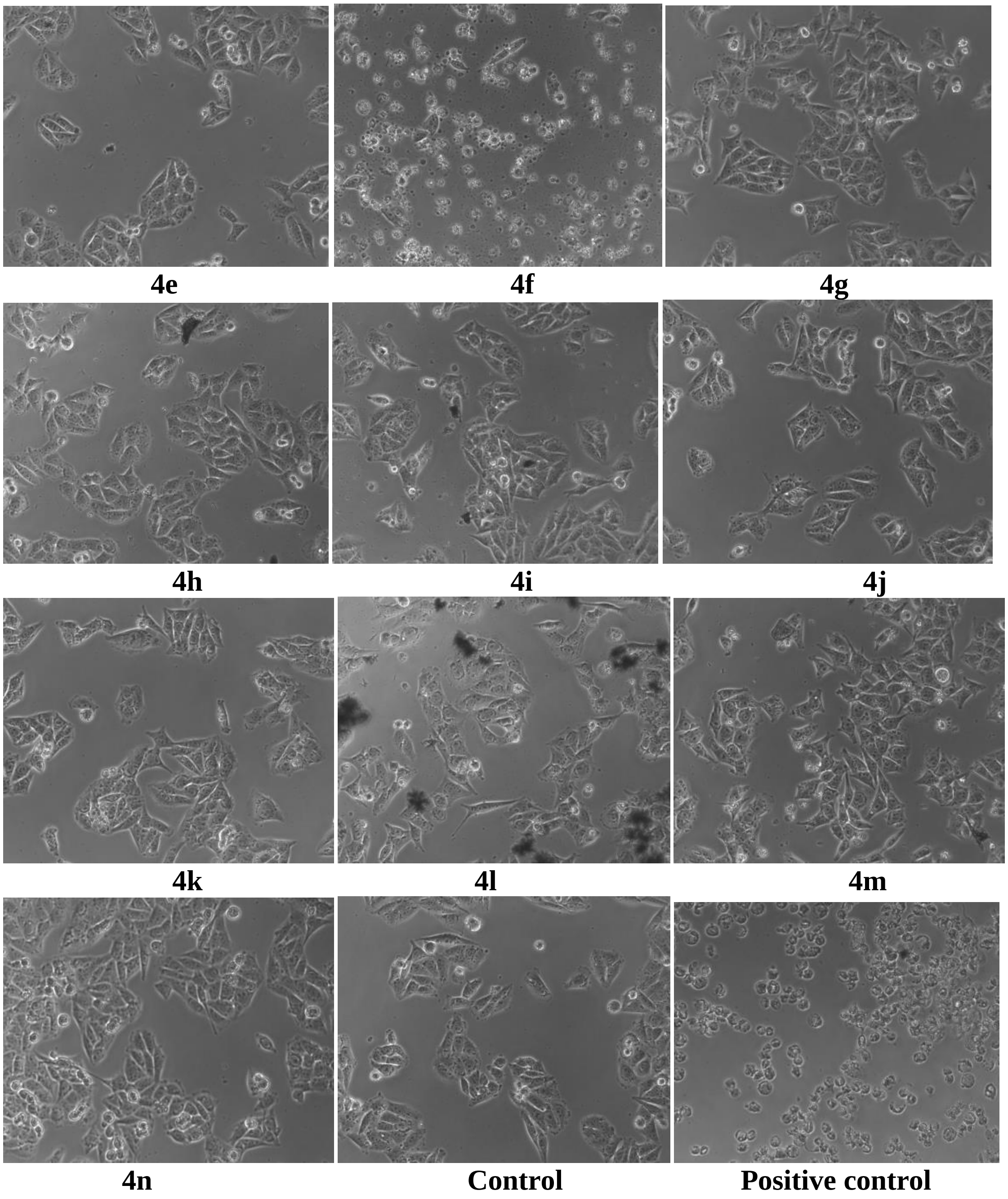

Figure S3. Images for *in-vitro* anticancer activity against IMR-32 cell line of the synthesized compounds **4(a-n),** control and positive control.

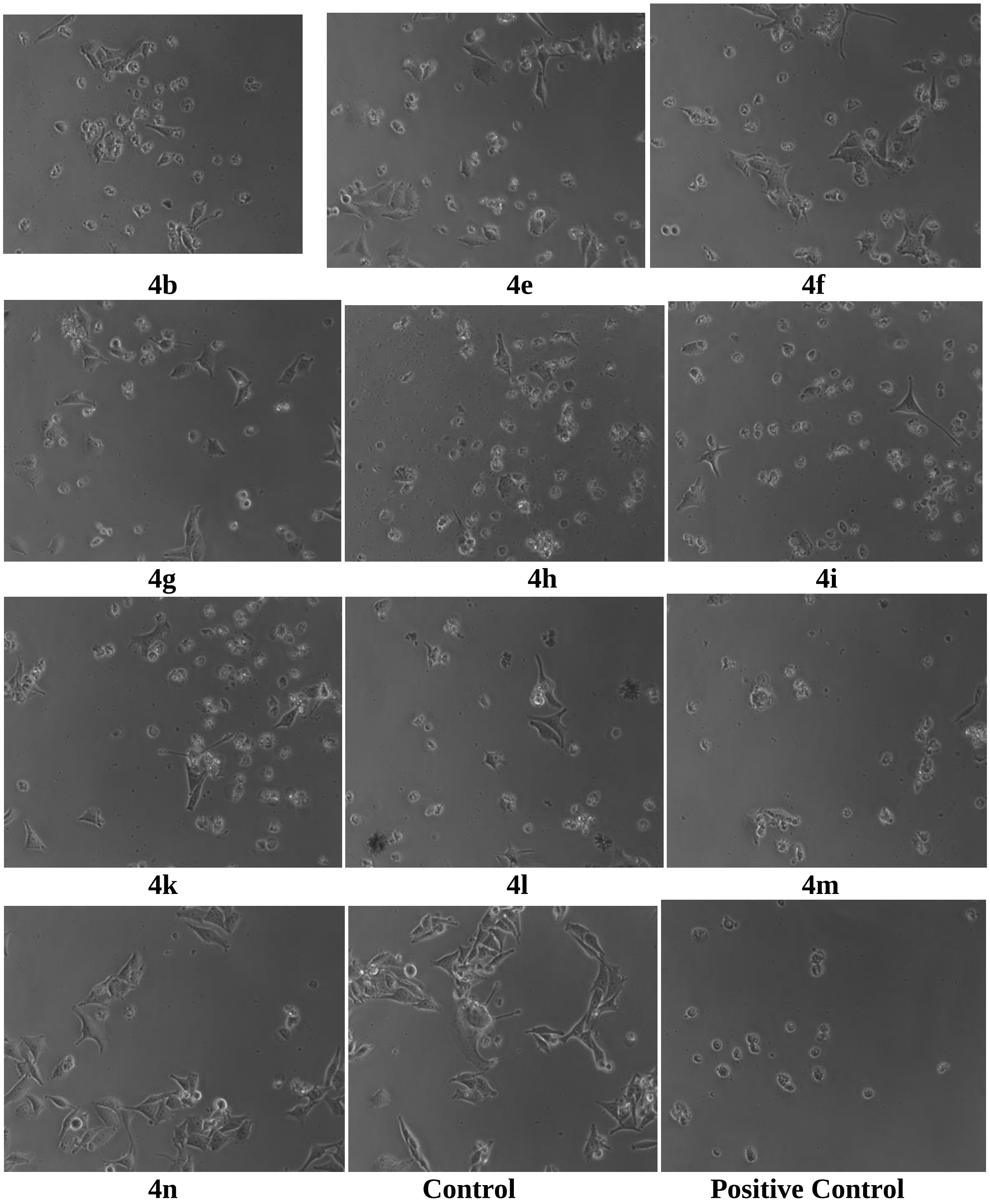

Figure S4. Images for *in-vitro* anticancer activity against SK-MEL-2 cell line of the synthesized compounds **4(a-n),** control and positive control.

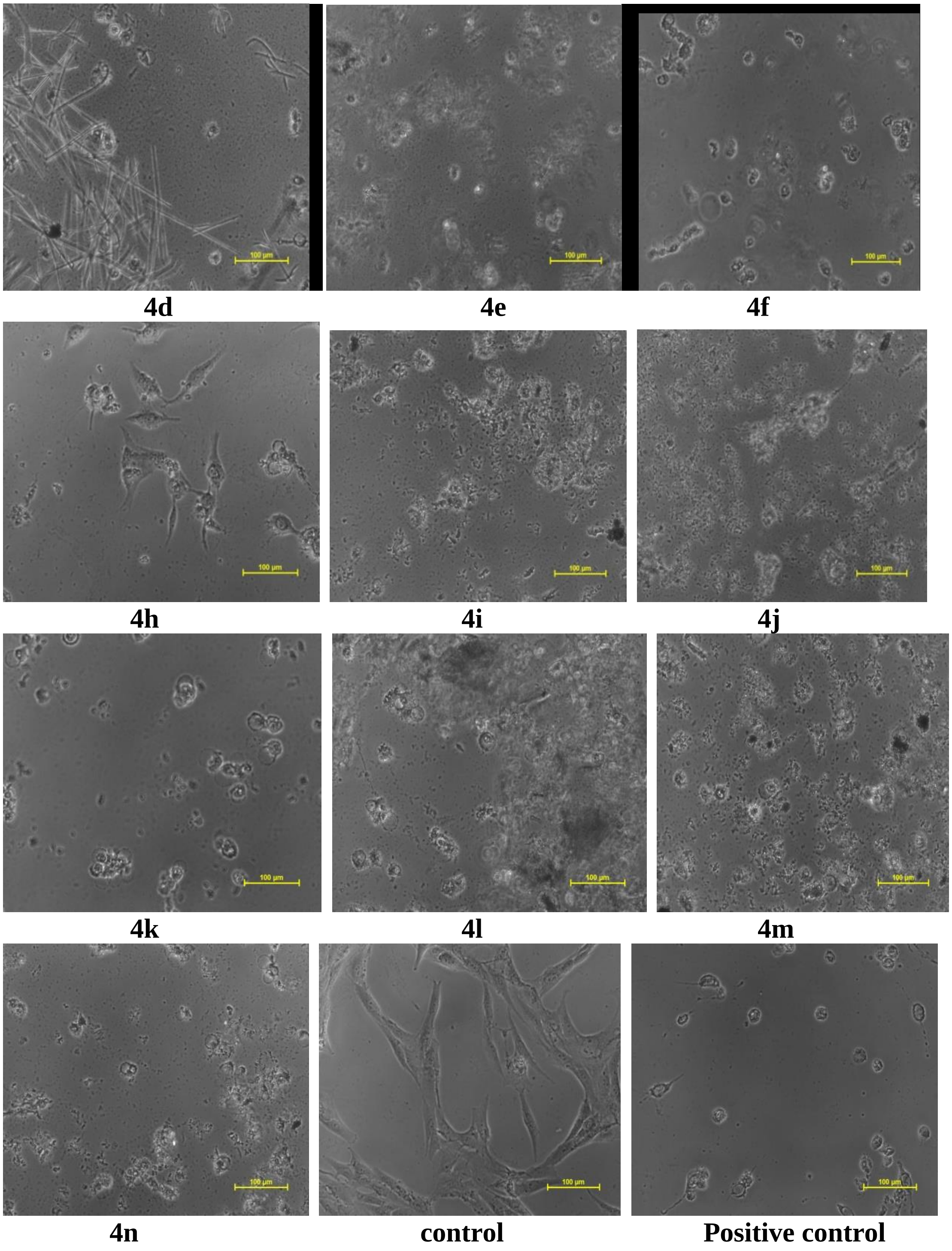

Figure S5. Images for *in-vitro* anticancer activity against MG-63 cell line of the synthesized compounds **4 (a-n)**, control and positive control.

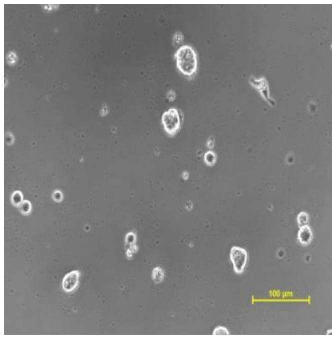 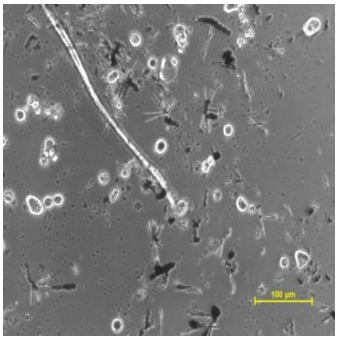 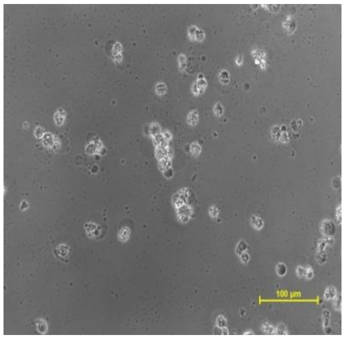

4d **4e** **4f**

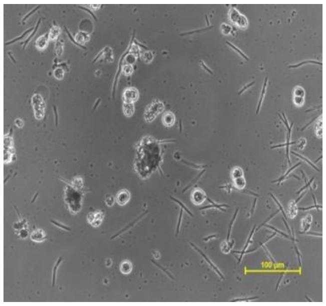 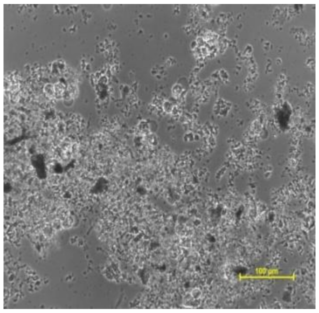 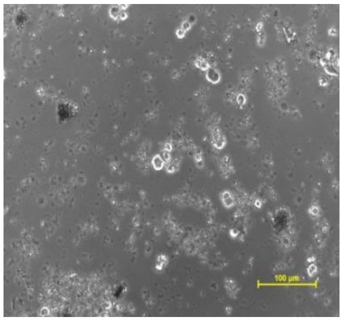

4h **4i** **4j**

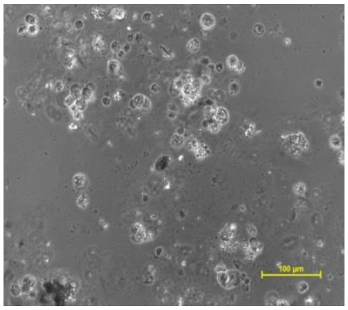 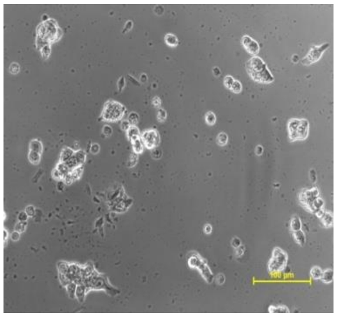 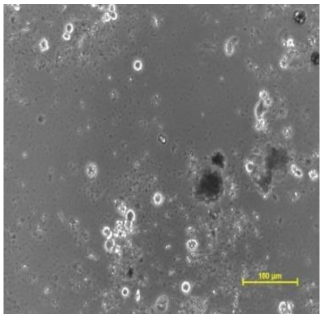

4k **4l** **4m**

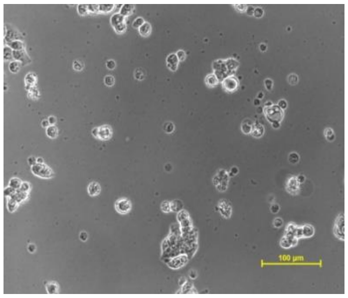 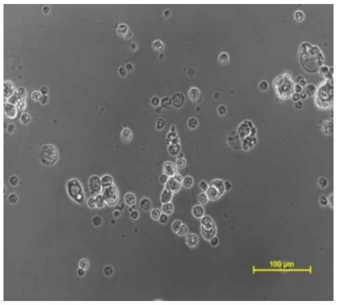

Control **positive control**

Figure S6. Images for *in-vitro* anticancer activity against HT-29 cell line of the synthesized compounds **4(a-n),** control and positive control.

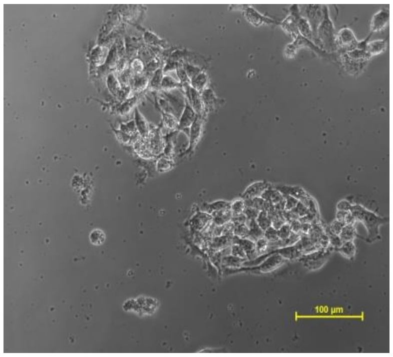 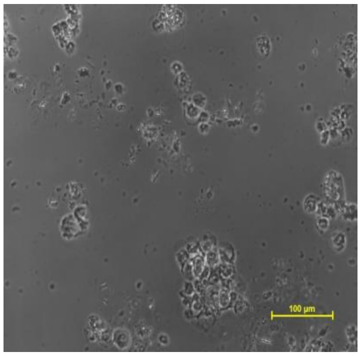 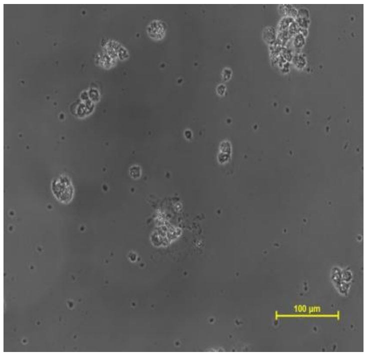

4c **4e** **4f**

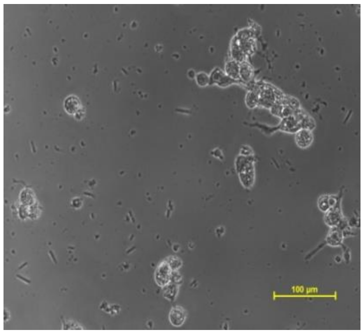 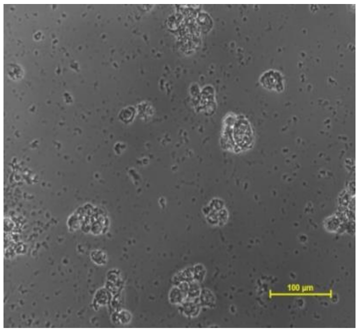 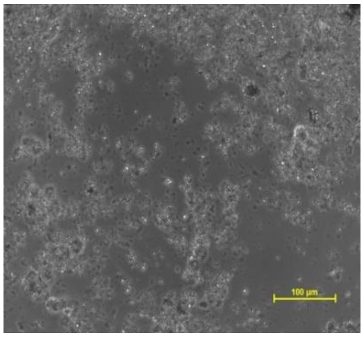

4g **4i** **4j**

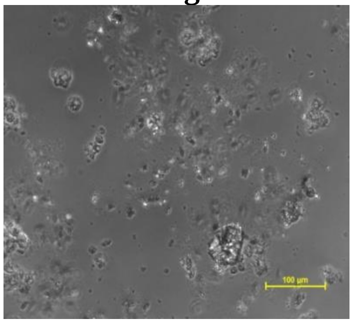 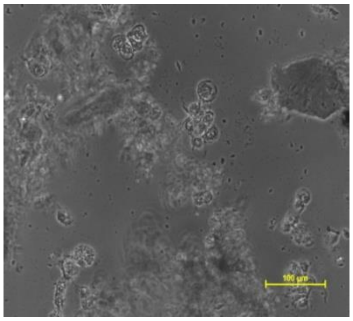 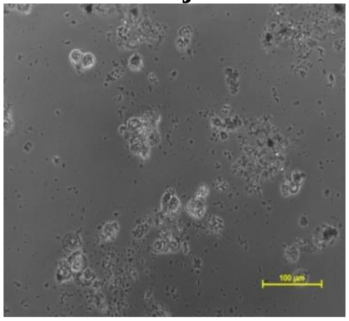

4k **4l** **4m**

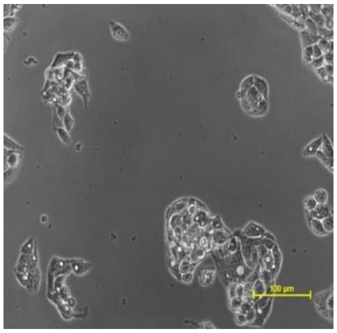 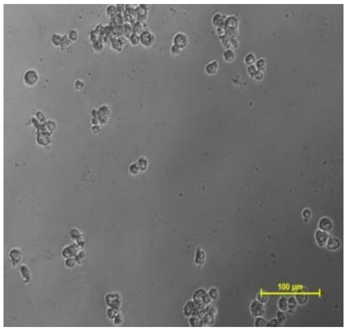

Control **positive control**

Figure S7. Images for *in-vitro* anticancer activity against Hep-G2 cell line of the synthesized compounds **4(a-n),** control and positive control.

^{1}HNMR spectrum of compound 4b

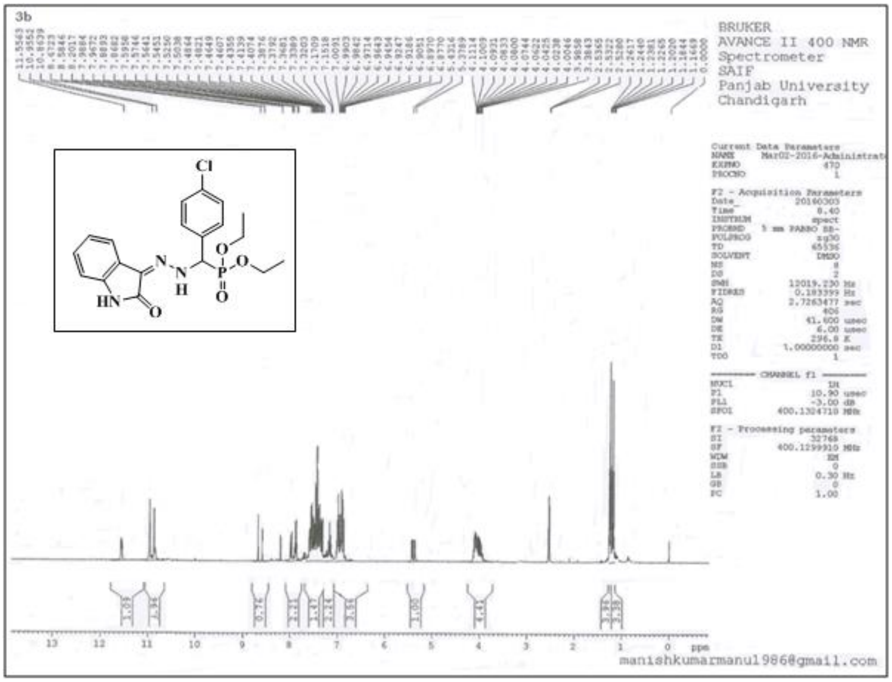

[13] CNMR spectrum of compound 4b

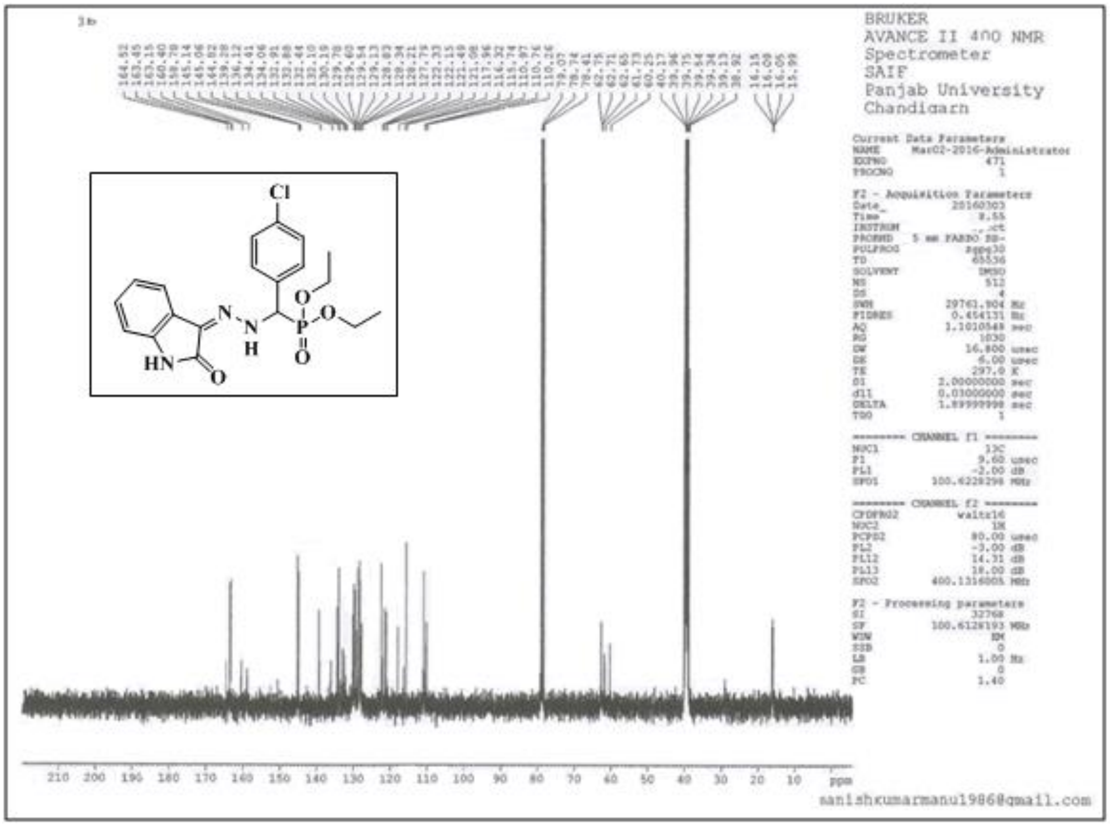

Mass spectra of compound 4b

Molecular Weight: 421.81, Molecular ion peak: 422.33

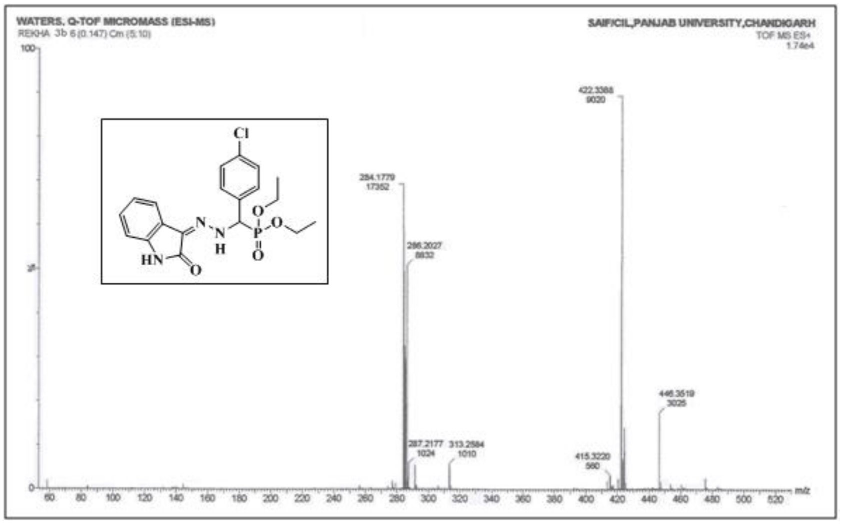

^{31}P NMR spectrum of compound 4b

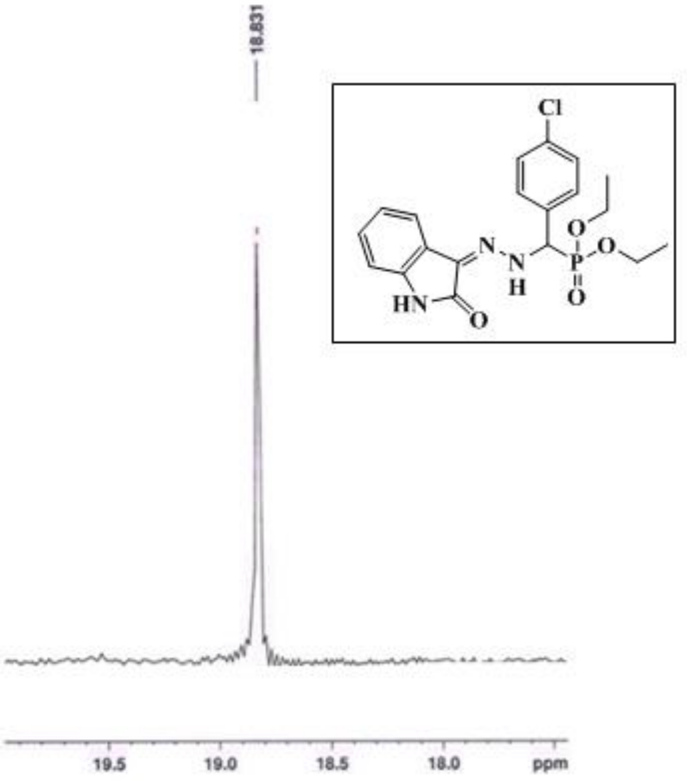

^{1}H NMR spectrum of 4d

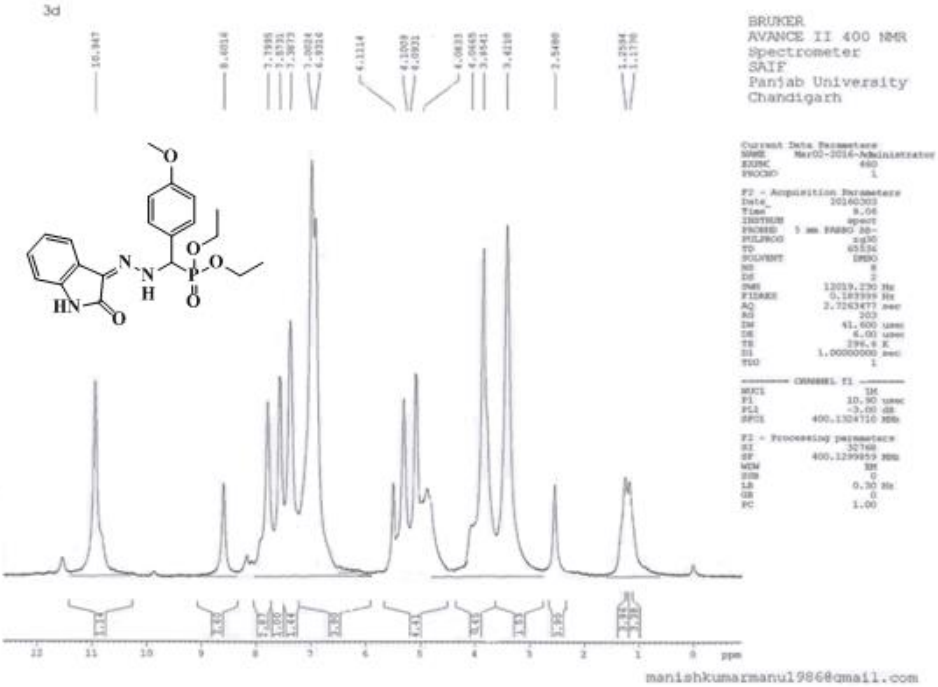

Mass spectra of 4d

Molecular Weight: 417.40, Molecular ion peak: 418.42.

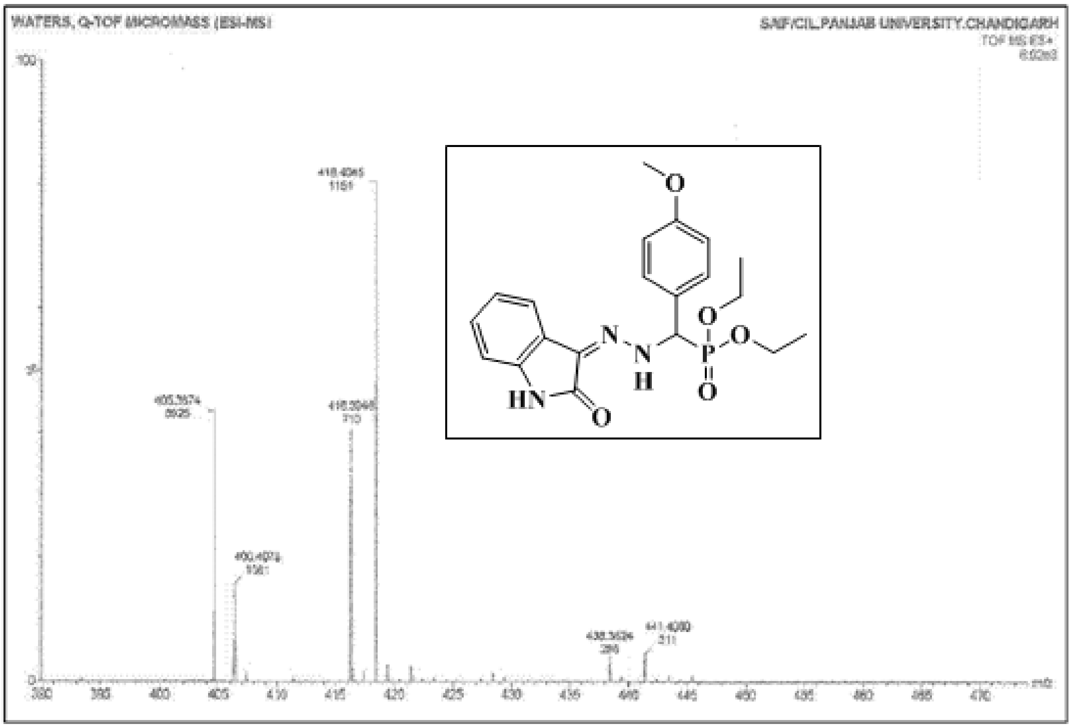

IR spectra of 4g

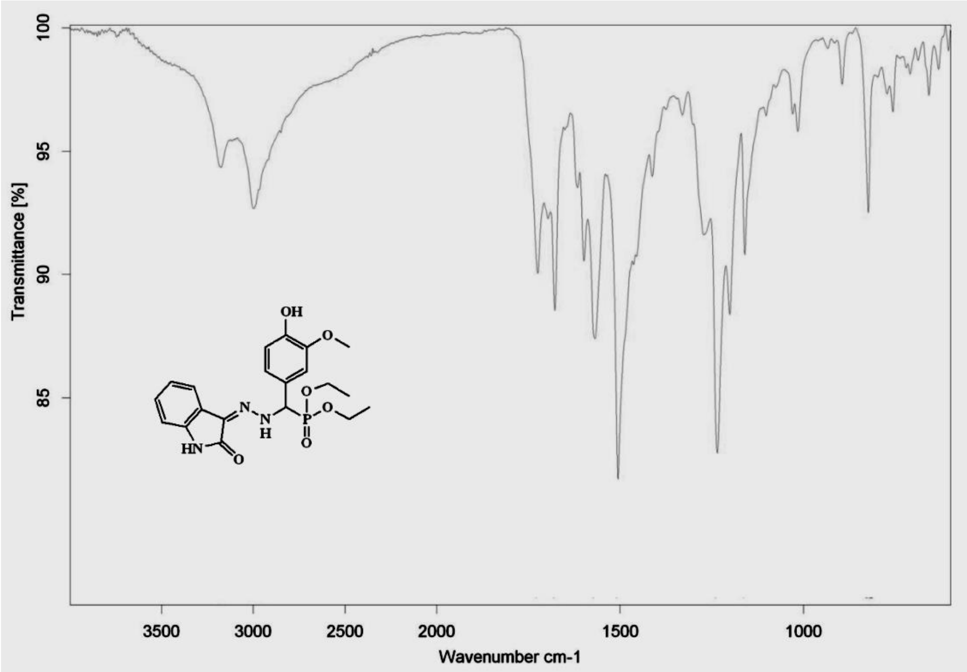

Elemental analysis of compound 4g

Method name:	CHNS
Analysed:	05/18/2017 13:00
Printed:	05-18-2017 17:10
Sample ID:	18May17016
Analysis type:	UnkNown
Chromatogram filename:	C:[redacted]18May17R1.DAT

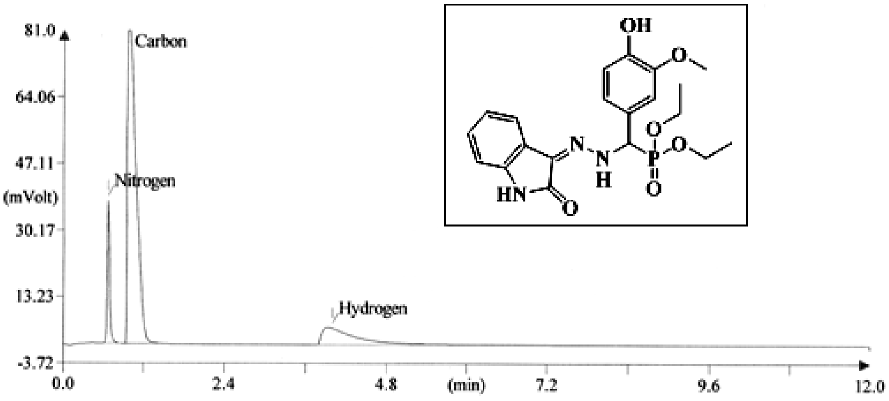

Component Name	Retention Time (min)	Area (.1*uV*sec)	Element %
Nitrogen	0.658	1048085	9.758
Carbon	0.975	6991914	55.490
Hydrogen	3.942	1499294	5.512
		9539293	70.760
